# Regulatory Roles of Small Non-coding RNAs in Sugar Beet Resistance Against *Beet curly top virus*

**DOI:** 10.3389/fpls.2021.780877

**Published:** 2022-01-10

**Authors:** Rajtilak Majumdar, Paul J. Galewski, Imad Eujayl, Rakesh Minocha, Eric Vincill, Carl A. Strausbaugh

**Affiliations:** ^1^Northwest Irrigation and Soils Research, United States Department of Agriculture-Agricultural Research Service, Kimberly, ID, United States; ^2^Northern Research Station, United States Department of Agriculture Forest Service, Durham, NH, United States

**Keywords:** BCTV (*Beet curly top virus*), sugar beet, RNAi, KDH13, KDH4-9, virus, resistance

## Abstract

*Beet curly top virus* (BCTV) mediated yield loss in sugar beets is a major problem worldwide. The circular single-stranded DNA virus is transmitted by the beet leafhopper. Genetic sources of BCTV resistance in sugar beet are limited and commercial cultivars rely on chemical treatments versus durable genetic resistance. Phenotypic selection and double haploid production have resulted in sugar beet germplasm (KDH13; 13 and KDH4-9; 4) that are highly resistant to BCTV. The molecular mechanism of resistance to the virus is unknown, especially the role of small non-coding RNAs (sncRNAs) during early plant–viral interaction. Using the resistant lines along with a susceptible line (KDH19-17; 19), we demonstrate the role of sugar beet microRNAs (miRNAs) in BCTV resistance during early infection stages when symptoms are not yet visible. The differentially expressed miRNAs altered the expression of their corresponding target genes such as pyruvate dehydrogenase (EL10Ac1g02046), carboxylesterase (EL10Ac1g01087), serine/threonine protein phosphatase (EL10Ac1g01374), and leucine-rich repeats (LRR) receptor-like (EL10Ac7g17778), that were highly expressed in the resistant lines versus susceptible lines. Pathway enrichment analysis of the miRNA target genes showed an enrichment of genes involved in glycolysis/gluconeogenesis, galactose metabolism, starch, and sucrose metabolism to name a few. Carbohydrate analysis revealed altered glucose, galactose, fructose, and sucrose concentrations in the infected leaves of resistant versus susceptible lines. We also demonstrate differential regulation of BCTV derived sncRNAs in the resistant versus susceptible lines that target sugar beet genes such as LRR (EL10Ac1g01206), 7-deoxyloganetic acid glucosyltransferase (EL10Ac5g12605), and transmembrane emp24 domain containing (EL10Ac6g14074) and altered their expression. In response to viral infection, we found that plant derived miRNAs targeted BCTV capsid protein/replication related genes and showed differences in expression among resistant and susceptible lines. The data presented here demonstrate the contribution of miRNA mediated regulation of metabolic pathways and cross-kingdom RNA interference (RNAi) in sugar beet BCTV resistance.

## Introduction

Sugar beet (*Beta vulgaris* L.) is a highly valuable crop that contributes to 55–60% of total sugar produced in the United States and Europe. Sugar yield from sugar beet is significantly reduced due to biotic stress resulting primarily from viral and fungal pathogens (Strausbaugh et al., 2011, 2013, 2017). Among viral pathogens, *Beet curly top virus* (BCTV) is one of the major causes of reduction in sugar beet yield and sugar production in semi-arid regions of the United States and globally (Srivastava, 2004; Panella et al., 2014). BCTV is a circular single-stranded DNA virus which belongs to the genus *Curtovirus* and is transmitted by the beet leafhopper (*Circulifer tenellus* Baker). Besides sugar beet, the virus can infect over 300 species within 44 plant families with significant impacts on crops like common beans, pepper, tomato, spinach, cucumber, etc. (Bennett, 1971). Sugar beet production was highly impacted by BCTV in the 1920s and early 1930s until resistant varieties were introduced (Bennett, 1971; Panella et al., 2014; Strausbaugh et al., 2017). The most economical control of BCTV in sugar beet would be through resistant cultivars though, most commercial cultivars possess only low to moderate resistance (Strausbaugh et al., 2017). As BCTV resistance is quantitatively inherited, the trait is challenging to maintain in the parental lines used for commercial hybrid production (Strausbaugh et al., 2007; Panella et al., 2014). Additional approaches such as seed and foliar treatments with synthetic insecticides have been successful in reducing sugar beet BCTV symptoms (Strausbaugh et al., 2010, 2012, 2014), additional sources of strong genetic resistance are highly desirable to develop eco-friendly management. Implementation of alternative cutting-edge molecular biology tools such as RNA interference (RNAi) mediated Host-Induced-Gene-Silencing (reviewed in Majumdar et al., 2017) will largely depend upon the identification of appropriate pathogen/host target genes and/or regulatory mechanisms that are highly critical during early stages of infection.

Small non-coding RNAs (sncRNAs) in this regard have been shown to play critical roles during host–pathogen interaction and therefore could serve as potential targets to improve host plant resistance (reviewed in Yang et al., 2021). Among different types of known sncRNAs, microRNAs (miRNAs) are highly conserved in plants and widely present in other species including animals and some viruses. They are approximately 22 nucleotides (nt) long and involved in RNA silencing at the post-transcriptional level via base-pairing with their target mRNAs at complementary regions followed by degradation of target transcripts. miRNAs resemble small interfering RNAs (siRNAs) related to RNAi, except the former is produced from transcripts containing inverted repeat sequences that form hairpin (hp) structures whereas the later originate from long double stranded RNAs. Both miRNAs and siRNAs are endogenous or exogenous to a living system depending upon interactions between living organisms. MicroRNAs modulate gene expression and regulate growth and development and response to stresses in plants and animals. Depending upon the nature of sequence complementarities between miRNAs and target transcripts, a specific miRNA can have multiple targets and a specific target gene can be regulated by multiple miRNAs (Riolo et al., 2020; Xu et al., 2020).

Cross-kingdom RNAi meditated by pathogen derived sncRNAs play an important role in down-regulating expression of key host defense related genes, especially during plant–virus interaction (reviewed in Ramesh et al., 2021). Host plants on the other hand, produce sncRNAs (e.g., miRNAs) that can modulate the expression of host metabolism or defense related genes besides targeting critical pathogenesis related genes (reviewed in Liu et al., 2017). Functional characterization of BCTV genes and their role in pathogenesis have been demonstrated in earlier studies. The *capsid protein* (*CP*), *C4*, and *C2* genes were shown to play a key role in viral pathogenesis and development of disease symptoms in plants. Mutation of the *CP* gene resulted in the loss of infectivity and the spread of the virus (Briddon et al., 1989). Similar mutational studies involving the *C4* gene demonstrated the role of this gene in virus movement and development of disease symptoms in plants (Teng et al., 2010). In contrast, the *C2* gene has been implicated in viral replication and creating a favorable cell environment for viral spread within host plants (Caracuel et al., 2012). No information is available on the role of BCTV-derived sncRNAs in pathogenicity during early stages of sugar beet infection. How plant-derived miRNAs may contribute to BCTV resistance by modulating host metabolic pathways and targeting key viral genes is also unknown.

In this study, we took a comprehensive approach to understand the regulatory role of sncRNAs in sugar beet resistance against BCTV and virus-derived sncRNAs in pathogenicity during early infection stages. Using a BCTV susceptible (S) sugar beet line (KDH19-17; 19) and two resistant (R) lines, KDH13 (13) and KDH4-9 (4), we demonstrate the role of sncRNAs in BCTV resistance/susceptibility. The line 19 is a double haploid and is highly susceptible to BCTV (Eujayl et al., 2016) and originated from the parental line C5944 (PI663873). The R lines (13 and 4) are also double haploids and highly resistant to BCTV (Eujayl et al., 2016) and were derived from the parental line C762-17 (PI 560130). We further validated virus sncRNA target genes in sugar beet and sugar beet miRNA target genes in sugar beet during cross-kingdom RNAi (sugar beet-BCTV), using global mRNAseq to analyze target gene expression. Our work for the first time demonstrates differential regulation of sugar beet miRNAs modulating host–plant metabolic pathways/defense-related genes and its contribution toward BCTV resistance and/or susceptibility.

## Materials and Methods

### Plant Growth, Viral Infection, and Sample Collection

Sugar beet plants at the 5–6 leaf-stage inside cages were exposed to beet leafhoppers (BLH) by releasing viruliferous BLHs (approximately 6–8 BLH/plant) carrying predominantly the BCTV Severe (BCTV-Svr) and BCTV CA/Logan strains. The uninfected control plants were similarly transferred into cages but without any BLHs. Growth chamber conditions were 28°C (day)/21°C (night), 16 h (day)/8 h (night) photoperiod, and 20% relative humidity. Newly emerged leaves from both control (uninfected) and BLH infected plants were collected at 1, 2, and 6 day post inoculation (dpi), flash frozen in liquid N, and stored at –80°C until further use. After sample collections at each time point, the plants were sprayed with Admire^®^ Pro (Bayer CropScience LLC, NC, United States) insecticide to eliminate any BLHs and future pest infections. This was followed by spraying with water to remove any residual insecticide, and then moved to the green house for symptom development in the infected plants in the subsequent weeks. The plants were evaluated for curly top symptoms at 3 week post inoculation.

### RNA Extraction, Construction of sRNA and mRNA Libraries, and Sequencing

Total RNA was extracted using “Plant/Fungi Total RNA Purification Kit” (Norgen Biotek Corp, ON, Canada) according to the manufacturer’s protocol. The quality and quantity of total RNA were analyzed using Bioanalyzer 2100 (Agilent Technologies, CA, United States) and acceptable RIN number for samples >7.0. High quality RNA was used for sRNAseq and mRNAseq library preparations. For sRNA libraries, approximately 1 μg of total RNA was used according to the TruSeq Small RNA Sample Prep Kit (Illumina, San Diego, CA, United States). This was followed by 50 bp single-end sequencing on an Illumina Hiseq 2500 sequencing platform at the LC Sciences (Houston, TX, United States) following the vendor’s protocol.

Poly(A) RNA sequencing libraries were prepared according to the Illumina’s TruSeq-stranded-mRNA sample preparation protocol. Approximately 1 μg of total RNA was used for this purpose. Depletion of ribosomal RNA were performed following the protocol described in the Ribo-Zero™ rRNA Removal Kit (Illumina, San Diego, CA, United States). Poly(A) mRNAs were purified using oligo-(dT) magnetic beads following two rounds of purification. Fragmentation of poly(A) RNA was performed using divalent cation buffer under elevated temperature. Cleaved RNA fragments were then reverse-transcribed to produce cDNA that was used to produce U-labeled second strand DNA. Following end repair, 3′ adenylation, adapter ligation, and PCR, final libraries were produced. Quality control and quantification of the sequencing libraries were done using Bioanalyzer 2100 (Agilent Technologies, CA, United States). Paired-end (150 bp) sequencing was performed on Illumina’s NovaSeq 6000 sequencing platform.

### Bioinformatics Analysis of miRNA

Raw reads were processed using an in-house program, ACGT101-miR (LC Sciences), that removed sequences associated with adapter dimers, common RNA families [ribosomal RNA (rRNA), transfer RNA (tRNA), small nuclear RNA (snRNA), small nucleolar RNA (snoRNA)], low quality reads, and repeats. Unique sequences of 18--25 bp in length were mapped to specific species precursors in the miRBase 22.0 database^1^ using BLAST to identify known miRNAs and novel miRNAs. The alignment took into consideration any length variation at both 3′ and 5′ ends and one mismatch inside the sequence. Known miRNAs were identified as sequences mapping to specific species mature miRNAs in the hairpin arms. The sequences mapping to the other arm of known specific species precursor hairpin opposite to the annotated mature miRNA-containing arm, were considered as novel 5p- or 3p derived miRNA candidates. The remaining sequences were mapped to other selected species precursors (with the exclusion of specific species) in the miRBase 22.0 database using BLAST search, and the mapped pre-miRNAs were further BLAST searched against the sugar beet genome to determine genomic locations. The unmapped sequences were BLAST searched against the sugar beet genome, and the hairpin RNA structures containing sequences were predicted from the flanking 120 nt sequences using the software RNAfold.^2^ A global normalization method was used to rectify copy numbers among different samples (Li et al., 2016). This helps to overcome any effect resulting from sequencing discrepancy on miRNA expression among samples. miRNAs that showed *p*-value <0.05 and fold change ≥1.5, were designated as Differentially Expressed (DE) miRNAs. Genes targeted by most abundant miRNAs was performed using computational target prediction algorithm PsRobot 1.2 (Wu et al., 2012). Predicted miRNA target genes were further validated using mRNAseq data by comparing expression of specific miRNAs to the expression of corresponding target genes.

### Read Mapping and Transcriptome Assembly

Raw reads originating from mRNA-Seq data were processed using in house (LC Sciences) Perl scripts and Cutadapt (Martin, 2011) to remove any low-quality reads and adapter sequences. FastQC^3^ was performed to analyze sequence quality. Reads were mapped to the sugar beet (*Beta vulgaris* subsp. *vulgaris*) reference genome EL10^4^ using Hisat2 (Kim et al., 2015) and mapped reads of each sample were assembled using StringTie (Pertea et al., 2015).

### Differentially Expressed mRNAs and Bioinformatics Analysis

All transcriptome data originating from different samples were merged to reconstruct a comprehensive transcriptome data using Perl scripts. Following merging, StringTie (Pertea et al., 2015) and edgeR (Robinson et al., 2010) software were used to estimate the expression levels of transcripts. Expression level of mRNAs was performed by calculating FPKM (fragments per kilobase of transcript per million mapped reads) using the StringTie software. EdgeR-R packages were used to estimate differential expression. A cutoff *p*-value of <0.05 and | log2 (fold-change)| > 1 was set to identify differentially expressed genes (DEGs).

Annotation of transcripts were performed using Blastx against the NCBI database. For Gene Ontology (GO) analysis, transcripts were blasted to the GO database to calculate the gene numbers for each term. Pathway enrichment was performed using the Kyoto Encyclopedia of Genes and Genomes (KEGG; Kanehisa et al., 2008).

### Analysis of Virus Derived sncRNAs

RNAseq reads, detailed previously, were aligned to a single consensus sequence from four BCTV genomes (Strausbaugh et al., 2017) which represent the important strains of BCTV in sugar beet: Severe strain CTS06-021 (GenBank accession KX867019.1), Colorado strain CTS15-113 (KX867056.1), CA/Logan strain CTS06-104 (KX867032.1) and the Worland strain CTS15-095 (KX867055.1). Alignment of reads was carried out using BWA (Li, 2013). The resulting files (.sam) were converted to (.bam) and sorted using Samtools v1.9 (Li et al., 2009). Only reads with high quality alignments (Q > 30) to the virus genome were retained for downstream analysis. These reads were converted to (.fasta) format. Each sequence name was derived from the sequence ID generated from the sequencer. The reads in (.fasta) format were reverse complemented using a short python script. The program blastn (Altschul et al., 1990) was carried out against the EL10.1 CDNA sequences. Blast results were parsed for information about core sequence homology of putative sncRNA species to the host sugar beet genome EL10.1. Specific sncRNA species were investigated further based on sequence length and percent identity between the core sncRNA species and their sugar beet genome targets. mRNA expression was evaluated for targets of sncRNA species by looking at FPKM values of the target genes. Using simple bash scripts to extract this information, statistical tests between treatments, and correlations between sncRNA abundance and gene expression (FPKM) of host target genes were carried out.

### Visualization of Virus Genome Transcription

The virus genome was visualized using the “R” software package. Virus gene positions were extracted from the Severe strain CTS06-021 (GenBank accession KX867019.1). Samtools depth was used to determine the coverage of reads across the genome for all time points. Read depth was plotted across the viral genome with respect to functional elements within the genome (e.g., viral genes and regions which produced specific sncRNA). Data were transformed into circular plots using functions found in the “Rcircos” source code (Zhang et al., 2013).

#### Plant miRNA Target Finding on the BCTV Genome

The miRNA sequences were reverse complemented using a python script (python rev_complement.py I_13.fa > I_13.fa_revcomp.fa). Blastn was used to search for homology of plant derived miRNA sequences to the genome of the BCTV-Svr strain CTS06-021 (GenBank accession KX867019.1) with the parameters in parentheses (blastn -db BCTVsevereseq.fa miRNA_revcomp.fa -outfmt 6 -word_size 18 -evalue 10 -num_alignments 5). mRNA expression for viral genes were estimated by alignment of the RNA-seq reads to the reference genome. HT-seq (Anders et al., 2014) was used to generate read counts for estimating the expression of viral genes.

### Evaluation of BCTV Symptoms and RT-PCR Analysis of BCTV Infected Sugar Beet Plants

Both uninfected control and BCTV inoculated plants at 1, 2, and 6 dpi were evaluated for any BCTV symptom development in the leaves and representative plants from each timepoint were photographed. Qualitative/quantitative analysis of viral load in the plant samples were determined through RT-PCR. cDNA was prepared using the iScript™ gDNA Clear cDNA Synthesis Kit (Bio-Rad, CA, United States) according to the manufacturer’s protocol. The thermocycler conditions included a pre-incubation step at 98°C for 30 s followed by 33 cycles of 98°C for 5 s (enzyme activation), 60°C for 10 s (primer annealing), 72°C for 3 s (elongation), and followed by a final elongation step at 72°C for 5 min. Equal amount of cDNA template was added to each sample for PCR amplification followed by gel electrophoresis of equal volume of the PCR products in a 2% agarose gel. The primers used for the amplification of BCTV capsid protein gene (*V1*), qCP_F-5′-TCCCAGAAAAGAAAGGTGAATCCT-3′ and qCP_R-5′-CCATTGGTATTTCCTCGAAGTCGT-3′, were universal for all 4 BCTV strains. The sugar beet house-keeping gene [glutamine synthetase (*GNL2*; EU558132.1)] primers were qBv_F-5′-CCTTCAGGGTGTCGCCAAT-3′ and qBv_F-5′-CCTTCAGGGTGTCGCCAAT-3′.

### Carbohydrate Analysis

Soluble sugars were extracted from approximately 100 mg of finely ground sugar beet leaf tissues (stored in –80°C) and analyzed by the method described here. Briefly, soluble sugars were extracted in 1 ml of 80% absolute ethanol at 65°C for 30 min. Samples were removed from heat, cooled for 5 min., then vortexed at medium speed for 2 min. and centrifuged at 13,000 rpm for 8 min to pellet tissue. Extracts were filtered through a 0.45 μm nylon syringe filter (Pall Corp., Port Washington, NY, United States) fitted onto a 3 ml syringe (Becton, Dickinson and Company, Franklin Lakes, NJ, United States). The profiles for 11 soluble sugars were determined using a PerkinElmer series 200 HPLC pump and autosampler (PerkinElmer Inc., Waltham, MA, United States) coupled with a Shimadzu RID-10A refractive index detector set at 30°C (Shimadzu Scientific Instruments Inc., Columbia, MD, United States). Sugars were separated on a Luna NH2 analytical column (heated to 25°C, 250 mm × 4.6 mm, 5 μm, 100 Å, Phenomenex Inc., Torrance, CA, United States) outfitted with an NH2 Guard column (4 mm × 3 mm, Phenomenex Inc.) using an isocratic mobile phase of 80% acetonitrile at a flow rate of 2 ml min^–1^. Besides Sucrose, other identified sugars in sugar beet leaves were quantified using a 5-point external standard curve (0.125–2 mg ml^–1^). For quantifying Sucrose, the standard curve was expanded from 2 to 18 mg ml^–1^. To quantify glucose + galactose (two peaks that did not separate*), the areas and concentrations of each were added together to create a combined standard curve. The chromatographs were analyzed, and the data were processed using PerkinElmer TotalChrom software (version 6.2.1).

### Data and Code Availability

The raw data resulting from sRNAseq (BioProject ID: PRJNA764694) and mRNAseq (BioProject ID: PRJNA764690) were submitted to the NCBI SRA database. All codes used in these steps are documented and available at ‘‘GitHub’’.^5^

## Results

### Sequencing Depth, Size Distribution, and Codon Biasedness of sncRNAs

**FIGURE 1:**
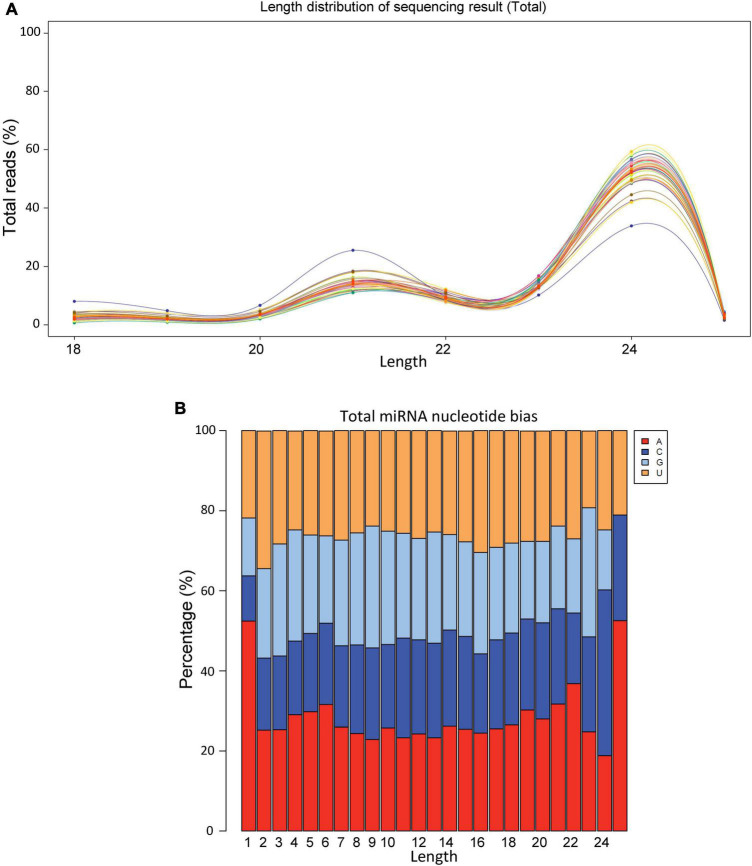
Size distribution and nucleotide bias of miRNAs derived from sugar beet leaves. **(A)** Size distribution and **(B)** nucleotide bias.

On an average we obtained >20 million raw reads/sample and >12 million valid reads/sample (Supplementary Table 1). The size distribution of sRNAs varied between 18 and 26 bp with the highest number of sRNAs were observed around 24 bp (Figure 1A). The nucleotide biasedness of sugar beet miRNAs revealed highest abundance of A, C, G, U at the nucleotide positions 1, 24, 23, and 2, respectively (Figure 1B).

### MicroRNAs Are Differentially Expressed in the S vs. R Lines at Early Infection Stages

**FIGURE 2:**
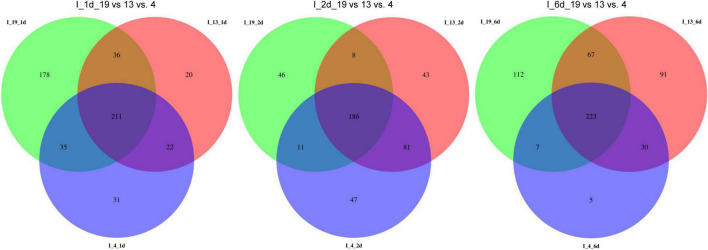
Venn diagrams of detected miRNAs in the apical leaves of virus infected (I) BCTV susceptible (Line 19; S) and resistant (Line 13 and Line 4; R) sugar beet lines at 1, 2, and 6 dpi.

**FIGURE 3:**
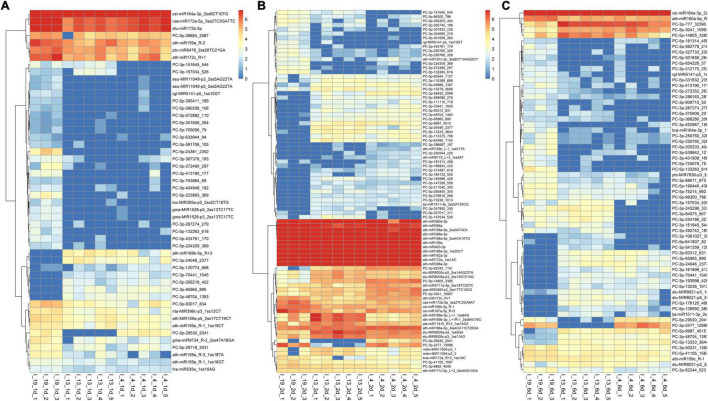
Heatmaps of differentially expressed (DE) miRNAs (*p* < 0.01) at **(A)** 1 dpi, **(B)** 2 dpi, and **(C)** 6 dpi in the leaves of BCTV susceptible (Line 19; S) and resistant (Line 13 and Line 4; R) sugar beet lines. Data are Mean ± SE of 3–5 biological replicates.

The total number of DE miRNAs that were unique to a specific line decreased from 1 to 6 dpi in the susceptible line 19 and in the resistant line 4 (Figure 2). Whereas, in the resistant line 13, the number of DE miRNAs increased over time. The changes observed in the control plants were relatively smaller compared to the infected plants (Supplementary Figure 1). Heatmaps of DE miRNAs (*p* < 0.01) at 1, 2, 6 dpi are shown in Figure 3 and the control samples in Supplementary Figure 2. All DE miRNAs (*p* < 0.05) in the infected and uninfected plants from the S and R lines are presented in Supplementary Tables 2–4. Examples of moderately expressed miRNAs at 1 dpi only in the R lines and absent in the S line are ath-miR169b-5p_R + 3, PC-3p-24046_2377, and PC-3p-85965_890 (Figure 3A and Supplementary Table 2). Some of the high to moderately expressed miRNAs that were down-regulated (>2-fold) only in the R lines (vs. S) are csi-miR164a-3p_2ss9CT10TG, ath-miR172c_R + 1, and PC-5p-93017_834. At 2 dpi, examples of miRNAs whose expression were high to moderate and were upregulated (∼1.5 to 50-fold) in the R lines (vs. S) are ath-miR166a-3p_1ss20CT, stu-MIR8005a-p5_1ss5GA, PC-5p-3041_10997, PC-5p-14805_3385, and PC-3p-62242_1151 (Figure 3B and Supplementary Table 3). Micro RNAs whose expression were high to moderate and were downregulated (∼2 to 30-fold) in the R lines (vs. S) are csi-miR167a-5p_R + 2, csi-miR156b-3p_R-1, and PC-5p-132263_616 to name a few. At 6 dpi, examples of miRNAs that were moderately expressed only in the R lines include PC-3p-93312_831 and PC-3p-85965_890 (Figure 3C and Supplementary Table 4). miRNAs whose expression were high to moderate and were upregulated (∼5 to 7-fold) in the R lines (vs. S) include PC-3p-777_32390, PC-5p-14805_3385, and PC-5p-3041_10997 to name a few. Examples of miRNAs that were down-regulated (∼2.5 to 27-fold) in the R lines (vs. S) are csi-miR164a-3p_2ss9CT10TG, ath-miR160a-5p_R-1, and PC-5p-205203_404.

### Gene Ontology and Kyoto Encyclopedia of Genes and Genomes Analyses of Differentially Expressed miRNAs

**FIGURE 4:**
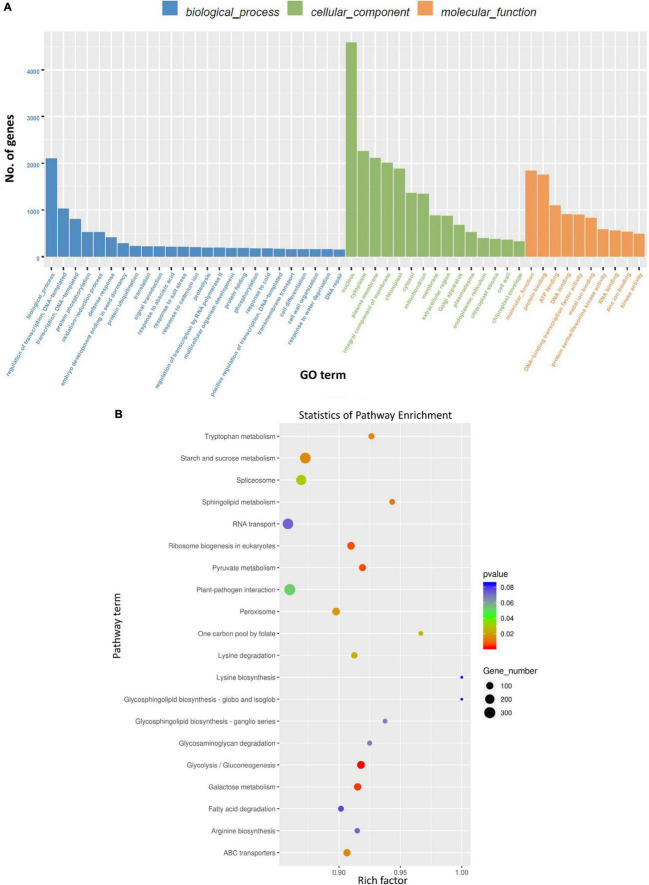
Gene ontology (GO) term and Kyoto Encyclopedia of Genes and Genomes (KEGG) enrichment of the sugar beet gene targets of differentially expressed (DE) sugar beet miRNAs in leaves. **(A)** GO annotation of sugar beet gene targets of DEmiRNAs in leaves, and **(B)** KEGG enrichment of sugar beet gene targets of DEmiRNAs.

Gene ontology (GO) analysis of genes targeted by DE miRNAs (considering all time points) due to BCTV infection represented genes that were mainly associated with cellular processes including transcription, protein phosphorylation, oxidation-reduction to name a few (Figure 4A). Pathway enrichment analysis of genes targeted by DE miRNAs (considering all time points) belonged to the pathways predominantly associated with glycolysis/gluconeogenesis, galactose metabolism, starch and sucrose metabolism, ribosome biogenesis, pyruvate metabolism etc. (Figure 4B and Supplementary Figure 3).

### Alteration of Expression of Sugar Beet Target Genes by Differentially Expressed miRNAs

We also analyzed potential sugar beet gene targets of DE sugar beet miRNAs. An extensive list of the target genes is presented in Supplementary Table 5. This list was further narrowed down to those target genes whose expression were altered by the DE miRNAs in the R vs. S lines. For mRNAseq, we obtained >32 million raw reads/sample and >31 million valid reads/sample (Supplementary Table 6). In several cases a single miRNA targeted multiple genes and reduced their expression. At 2 dpi, examples of miRNAs that were upregulated in the R lines (Table 1) and reduced target gene expression are lja-miR1511-3p_2ss5AT24CG targeting EL10Ac7g16579 (SPX domain-containing membrane protein; 11 to 20-fold downregulation), PC-5p-48966_1387 targeting EL10Ac6g14610 (cyclin-dependent kinase; 4 to 5-fold downregulation), stu-MIR8005a-p3_2ss15GT21AG targeting EL10Ac9g22557 (ubiquitin-like domain-containing CTD phosphatase; 2 to 400-fold downregulation), stu-MIR8005a-p5_1ss5GA targeting EL10Ac1g00203 (transcription factor bHLH14; 2 to 5-fold downregulation), and stu-MIR8005c-p5_2ss14AG23TA targeting EL10Ac2g03749 (transcription factor; >200-fold downregulation). At 2 dpi, some of the downregulated miRNAs in the R lines (Table 2) include ahy-miR167-3p_L-1_2_ss12CT15TC targeting EL10Ac4g09000 (hypothetical_protein; >20,000-fold upregulation), ath-miR396a-5p targeting EL10Ac5g11116 (chaperone protein; 2 to 3-fold upregulation), cas-miR172a-5p_3ss2_TC3GA9AT targeting EL10Ac3g05363 (ABC transporter; 4 to 4.5-fold upregulation), gma-MIR159e-p3_2ss16AG18TA targeting EL10Ac1g02046 (pyruvate dehydrogenase; >80,000-fold upregulation), and gma-MIR159e-p3_2ss16AG18TA targeting EL10Ac9g21768 (probable LRR receptor-like serine/threonine-protein kinase; ∼2-fold upregulation).

**TABLE 1:** Sugar beet miRNAs that showed >2-fold expression in the apical leaves of BCTV resistant (13, 4) vs. susceptible (19) lines at 2 dpi and effect on corresponding sugar beet target gene expression.

miRNA	Sequence	Gene ID	Gene annotation	miRNA abundance (FPKM)			Target gene expression (FPKM)			Fold change (FPKM)		Correlation coefficient
							
				Line 19	Line 13	Line 4	Line 19	Line 13	Line 4	Line 19 vs Line 13	Line 19 vs Line 4	
lja-miR1511-3p_2ss5AT24CG	AACCTGGCTCTGATACCATGAAGG	EL10Ac7g16579	SPX_domain-containing_membrane_protein_ At4g22990;TMhmm_ExpAA:216.46	0	9	10	3.9	0.6	0.2	11.0	20.0	–1.00
mdm-miR3627a_L + 1R + 1_1ss21AG	CTCGCAGGAGAGATGGCACTGG	EL10As7g23813	Probable_leucine-rich_repeat_receptor-like_protein_kinase_At5g49770; SignalP:0.771;TMhmm_ExpAA:26.55	154	312	419	3.9	1.5	1.8	2.6	2.2	–0.89
PC-3p-48704_1393	CGTTCGGGCCATGGGGCGATC	EL10Ac7g17584	Major_allergen_Pru_ar_1	1	14	19	0.3	0.0	0.4	2.5	1.2	–0.95
PC-3p-62242_1151	TTTCCCAACAAGTATCAGCATC	EL10Ac1g01335	Heat_shock_cognate_70_ kDa_protein	5	114	273	0.1	0.0	0.0	2.5	11.7	–0.95
PC-3p-62242_1151	TTTCCCAACAAGTATCAGCATC	EL10Ac4g10316	Multiple_C2_and_ transmembrane_domain-containing_protein_1;TMhmm_ ExpAA:72.50	5	114	273	1.0	1.4	1.3	2.1	1.2	–0.87
PC-3p-62242_1151	TTTCCCAACAAGTATCAGCATC	EL10Ac5g10901	Probable_mitochondrial_ chaperone_BCS1-B;TMhmm_ExpAA:20.62	5	114	273	0.0	0.0	0.1	6.9	523.6	–0.81
PC-3p-62242_1151	TTTCCCAACAAGTATCAGCATC	EL10Ac8g20102	Wall-associated_receptor_kinase _galacturonan-binding;SignalP:0.906; TMhmm_ExpAA:42.08	5	114	273	2.6	2.7	2.8	2.9	1.1	–0.85
PC-5p-278618_289	AGTTTTCGGATGATATAGTAGGGT	EL10Ac9g21069	O-acyltransferase_WSD1	0	11	8	0.0	0.0	0.7	1751.9	30.8	–0.97
PC-5p-3041_10997	GGTAAGAGATTGTTAAAAATT	EL10Ac2g03607	ABC_transporter_D _family_member_2,_chloroplastic; TMhmm_ExpAA:94.97	48	136	264	7.5	4.1	2.5	2.2	3.0	–0.91
PC-5p-3041_10997	GGTAAGAGATTGTTAAAAATT	EL10Ac4g08705	Chaperone_protein_ClpB1	48	136	264	4.0	4.6	6.0	2.2	1.5	–0.83
PC-5p-3041_10997	GGTAAGAGATTGTTAAAAATT	EL10Ac4g09537	G-type_lectin_S-receptor-like_serine/threonine-protein_kinase_RLK1; SignalP:0.93;TMhmm_ExpAA:55.30	48	136	264	1.7	0.5	0.5	2.4	3.4	–0.87
PC-5p-48966_1387	CATGGGGCGATCGTCCGGCCA	EL10Ac6g14610	Cyclin-dependent_kinase_C-1	5	27	31	0.4	0.1	0.1	4.4	4.9	–0.94
PC-5p-73236_1013	AATAGGGCTAAAAGTCGTTGC	EL10Ac1g00123	Multiple_C2_and_transmembrane_ domain-containing_protein_1; TMhmm_ExpAA:62.10	0	8	8	0.2	0.2	0.3	3.5	1.2	–0.97
PC-5p-73236_1013	AATAGGGCTAAAAGTCGTTGC	EL10Ac3g05165	Caffeic_acid_3-*O*-methyltransferase	0	8	8	15.6	7.7	13.0	2.1	1.2	–0.97
PC-5p-73236_1013	AATAGGGCTAAAAGTCGTTGC	EL10Ac5g10584	Testis-expressed_sequence_11_protein	0	8	8	1.0	0.6	0.3	2.9	3.4	–0.98
PC-5p-73236_1013	AATAGGGCTAAAAGTCGTTGC	EL10Ac8g20087	Probable_F-box_protein_At4g22030	0	8	8	0.0	0.0	0.0	163.9	1.0	–1.00
stu-MIR8005a-p3_2ss15GT21AG	TTTAGGGTTTAGGGTTTAGGGTA	EL10Ac4g10316	Multiple_C2_and_transmembrane_domain-containing_protein_1; TMhmm_ExpAA:72.50	36	202	121	1.0	1.4	1.3	2.1	1.2	–0.81
stu-MIR8005a-p3_2ss15GT21AG	TTTAGGGTTTAGGGTTTAGGGTA	EL10Ac5g12219	Beta-amylase_3,_chloroplastic	36	202	121	12.9	6.0	15.5	2.4	1.2	–1.00
stu-MIR8005a-p3_2ss15GT21AG	TTTAGGGTTTAGGGTTTAGGGTA	EL10Ac5g12363	3-isopropylmalate_dehydratase_small_subunit_3	36	202	121	18.4	6.0	8.8	2.3	2.1	–0.88
stu-MIR8005a-p3_2ss15GT21AG	TTTAGGGTTTAGGGTTTAGGGTA	EL10Ac9g22557	Ubiquitin-like_domain-containing_CTD_phosphatase	36	202	121	1.4	0.2	0.7	395.1	2.0	–0.87
stu-MIR8005a-p5_1ss5GA	GTTTAGGGTTTAGGGTTTAGGGTT	EL10Ac1g00203	Transcription_factor_bHLH14	98	645	315	15.6	3.1	8.0	4.7	2.0	–0.81
stu-MIR8005a-p5_1ss5GA	GTTTAGGGTTTAGGGTTTAGGGTT	EL10Ac5g12219	Beta-amylase_3,_chloroplastic	98	645	315	12.9	6.0	15.5	2.4	1.2	–1.00
stu-MIR8005a-p5_1ss5GA	GTTTAGGGTTTAGGGTTTAGGGTT	EL10Ac5g12363	3-isopropylmalate_dehydratase_small_subunit_3	98	645	315	18.4	6.0	8.8	2.3	2.1	–0.81
stu-MIR8005c-p5_2ss14AG23TA	TTTAGGGTTTAGGGTTTAGGGTA	EL10Ac2g03749	Transcription_factor_DIVARICATA	36	202	121	4.4	0.0	0.0	217.6	43798.0	–0.87
stu-MIR8005c-p5_2ss14AG23TA	TTTAGGGTTTAGGGTTTAGGGTA	EL10Ac4g10316	Multiple_C2_and_transmembrane_domain-containing_protein_1; TMhmm_ExpAA:72.50	36	202	121	1.0	1.4	1.3	2.1	1.2	–0.81
stu-MIR8005c-p5_2ss14AG23TA	TTTAGGGTTTAGGGTTTAGGGTA	EL10Ac5g12219	Beta-amylase_3,_chloroplastic	36	202	121	12.9	6.0	15.5	2.4	1.2	–1.00
stu-MIR8005c-p5_2ss14AG23TA	TTTAGGGTTTAGGGTTTAGGGTA	EL10Ac5g12363	3-isopropylmalate_dehydratase_small_subunit_3	36	202	121	18.4	6.0	8.8	2.3	2.1	–0.88
stu-MIR8005c-p5_2ss14AG23TA	TTTAGGGTTTAGGGTTTAGGGTA	EL10Ac9g22557	Ubiquitin-like_domain-containing_CTD_phosphatase	36	202	121	1.4	0.2	0.7	395.1	2.0	–0.87

*A strong negative correlation (<–0.80 to –1.00) indicates higher abundance of a specific sncRNA and lower expression of corresponding sncRNA target sugar beet gene/s. Data are Mean of 3–5 biological replicates.*

**TABLE 2:** Sugar beet miRNAs that showed < 2-fold expression in the apical leaves of BCTV resistant (13, 4) vs. susceptible (19) lines at 2 dpi and effect on corresponding sugar beet target gene expression.

miRNA	Sequence	Gene ID	Gene annotation	miRNA abundance (FPKM)			Target gene expression (FPKM)			Fold change (FPKM)		Correlation coefficient
							
				Line 19	Line 13	Line 4	Line 19	Line 13	Line 4	Line 13 vs Line 19	Line 4 vs Line 19	
ahy-miR167-3p_L-1_2_ss12CT15TC	GATCATGTGGTAGCTTCACC	EL10Ac3g05809	Disease_resistance_protein_RGA2	4473	1828	1703	0.2	0.5	1.6	11.8	12.0	–1.00
ahy-miR167-3p_L-1_2_ss12CT15TC	GATCATGTGGTAGCTTCACC	EL10Ac4g09000	hypothetical_protein	4473	1828	1703	0.0	2.3	2.6	26979.3	30574.5	–1.00
ahy-miR167-3p_L-1_2_ss12CT15TC	GATCATGTGGTAGCTTCACC	EL10Ac7g17047	Cationic_peroxidase_1;SignalP:0.591	4473	1828	1703	0.9	2.6	3.3	4.0	2.5	–0.84
ath-miR396a-5p	TTGGCATTCTGTCCACCTCC	EL10Ac5g11116	Chaperone_protein_ClpB1	1636	806	918	2.7	10.9	8.5	2.8	3.2	–0.96
ath-miR396a-5p	TTGGCATTCTGTCCACCTCC	EL10Ac8g18328	Oxysterol-binding_protein-related_protein_4C	1636	806	918	0.0	0.5	1.3	34.9	48.8	–0.92
cas-miR172a-5p_3ss2_TC3GA11CT	GCAGCATCATTAAGATTCACA	EL10Ac2g02633	Putative_disease_resistance_RPP13-like_protein_1	206	65	80	0.0	0.5	0.8	8761.2	5436.2	–0.96
cas-miR172a-5p_3ss2_TC3GA11CT	GCAGCATCATTAAGATTCACA	EL10Ac3g05348	hypothetical_protein	206	65	80	5.8	11.2	12.9	2.1	2.0	–1.00
cas-miR172a-5p_3ss2_TC3GA9AT	GCAGCATCTTCAAGATTCACA	EL10Ac3g05363	ABC_transporter_C_ family_member_10; TMhmm_ExpAA:300.24	423	118	156	3.7	17.7	14.6	4.5	4.1	–1.00
cas-miR172a-5p_3ss2_TC3GA9AT	GCAGCATCTTCAAGATTCACA	EL10Ac3g05812	Probable_carboxylesterase_1	423	118	156	4.1	8.6	11.5	2.4	2.5	–0.98
cas-miR172a-5p_3ss2_TC3GA11CT	GCAGCATCATTAAGATTCACA	EL10Ac4g09232	hypothetical_protein	206	65	80	4.3	8.1	7.8	2.4	2.7	–0.97
cas-miR172a-5p_3ss2_TC3GA11CT	GCAGCATCATTAAGATTCACA	EL10Ac7g15913	Malonyl-CoA-acyl_carrier_protein_transacylase,_mitochondrial	206	65	80	23.0	51.2	45.3	2.4	2.1	–1.00
gma-MIR159e-p3_2ss16AG18TA	AGCTCCTTGAAGTCCGAAT	EL10Ac1g01087	Probable_carboxylesterase_18	90	24	19	0.0	2.0	3.5	22162.7	38477.0	–0.93
gma-MIR159e-p3_2ss16AG18TA	AGCTCCTTGAAGTCCGAAT	EL10Ac1g02046	Pyruvate_dehydrogenase_E1_component_subunit_ beta,_mitochondrial	90	24	19	0.0	9.0	8.5	88279.9	84113.1	–0.99
gma-MIR159e-p3_2ss16AG18TA	AGCTCCTTGAAGTCCGAAT	EL10Ac7g17862	Small_ubiquitin-related_modifier_2	90	24	19	8.7	13.5	21.2	2.8	3.4	–0.98
gma-MIR159e-p3_2ss16AG18TA	AGCTCCTTGAAGTCCGAAT	EL10Ac9g21768	Probable_LRR_receptor-like_serine/threonine-protein_kinase_At3g47570; TMhmm_ExpAA:121.10	90	24	19	1.3	2.3	4.6	2.2	1.9	–0.93

*A strong negative correlation (<–0.80 to –1.00) indicates higher abundance of a specific sncRNA and lower expression of corresponding sncRNA target sugar beet gene/s. Data are Mean of 3–5 biological replicates.*

Some of the upregulated miRNAs in the R lines at 6 dpi (Table 3) that targeted sugar beet genes include PC-3p-48704_1393 targeting EL10Ac5g12315 (ethylene-responsive transcription factor; 1.5 to 4-fold downregulation), PC-3p-70441_1045 targeting EL10Ac5g12641 (long chain acyl-CoA synthetase; >70-fold downregulation), PC-3p-777_32390 targeting EL10Ac9g21076 (putative disease resistance protein RGA4; >350-fold downregulation), and PC-5p-178120_466 targeting EL10Ac2g02731 (probable carboxylesterase; ∼2.5-fold downregulation). At 6 dpi, miRNAs that were downregulated in the R lines (Table 4) and targeted sugar beet genes include ath-miR160a-5p_R-1 targeting EL10Ac1g01085 (15-cis-phytoene desaturase; ∼370 to 450-fold upregulation), ath-miR160a-5p_R-1 targeting EL10Ac2g02632 (putative disease resistance RPP13-like protein; ∼15 to 25-fold upregulation), ath-miR160a-5p_R-1 targeting EL10Ac7g17778 (probable LRR receptor-like serine/threonine-protein kinase; 15 to 17-fold upregulation), bra-miR164e-3p_1ss14CT targeting EL10Ac2g02793 (putative disease resistance RPP13-like protein; ∼30-fold upregulation), PC-3p-50231_1360 targeting EL10Ac1g01374 (serine/threonine-protein phosphatase; >20,000-fold upregulation), PC-3p-539842_121 targeting EL10Ac6g14637 (heat shock protein; >35,000-fold upregulation), PC-5p-205203_404 targeting EL10Ac1g01085 (15-*cis*-phytoene desaturase; ∼370 to 450-fold upregulation), and PC-5p-725678_75 targeting EL10Ac8g20096 (LRR-like serine/threonine-protein kinase; ∼6 to 9-fold upregulation).

**TABLE 3:** Sugar beet miRNAs that showed > 4-fold expression in the apical leaves of BCTV resistant (13, 4) vs. susceptible (19) lines at 6 dpi and effect on corresponding sugar beet target gene expression.

miRNA	Sequence	Gene ID	Gene annotation	miRNA abundance (FPKM)			Target gene expression (FPKM)			Fold Change (FPKM)		Correlation coefficient
							
				Line 19	Line 13	Line 4	Line 19	Line 13	Line 4	Line 19 vs Line 13	Line 19 vs Line 4	
PC-3p-48704_1393	CGTTCGGGCCATGGGGCGATC	EL10Ac5g12315	Ethylene-responsive_transcription_factor_ERF105	4	27	24	108.1	30.1	80.1	3.6	1.3	–0.84
PC-3p-70441_1045	CTAGGAAGAGGGGTACTTTTGG	EL10Ac5g11384	Transcription_factor_TCP20	0	13	13	18.8	6.7	7.2	2.8	2.6	–1.00
PC-3p-70441_1045	CTAGGAAGAGGGGTACTTTTGG	EL10Ac5g12279	Expansin-like_B1;SignalP:0.781;TMhmm_ExpAA:17.28	0	13	13	2.0	1.0	1.3	2.1	1.5	–0.93
PC-3p-70441_1045	CTAGGAAGAGGGGTACTTTTGG	EL10Ac5g12641	Long_chain_acyl-CoA_synthetase_9,_chloroplastic	0	13	13	3.6	0.1	0.0	72.0	36272	–1.00
PC-3p-777_32390	TTTTTAACAATCTCTTTTCCAA	EL10Ac2g02916	Probable_mitochondrial_chaperone_BCS1-B	105	731	507	21.9	4.8	12.1	4.5	1.8	–1.00
PC-3p-777_32390	TTTTTAACAATCTCTTTTCCAA	EL10Ac4g07836	CRS2-associated_factor_2,_mitochondrial	105	731	507	8.5	3.6	3.9	2.4	2.2	–0.95
PC-3p-777_32390	TTTTTAACAATCTCTTTTCCAA	EL10Ac5g11384	Transcription_factor_TCP20	105	731	507	18.8	6.7	7.2	2.8	2.6	–0.95
PC-3p-777_32390	TTTTTAACAATCTCTTTTCCAA	EL10Ac5g11907	Calcium_homeostasis_endoplasmic_reticulum_protein	105	731	507	20.7	10.0	10.8	2.1	1.9	–0.96
PC-3p-777_32390	TTTTTAACAATCTCTTTTCCAA	EL10Ac7g17117	Tyrosyl-DNA_phosphodiesterase_1	105	731	507	3.4	1.3	2.0	2.6	1.7	–1.00
PC-3p-777_32390	TTTTTAACAATCTCTTTTCCAA	EL10Ac7g17642	Putative_disease_resistance_protein_RGA1;SignalP:0.485	105	731	507	2.4	0.0	0.0	23960	330.1	–0.94
PC-3p-777_32390	TTTTTAACAATCTCTTTTCCAA	EL10Ac7g17945	DEAD-box_ATP-dependent_RNA_helicase_41	105	731	507	9.9	4.9	5.7	2.0	1.7	–0.98
PC-3p-777_32390	TTTTTAACAATCTCTTTTCCAA	EL10Ac8g20140	Fructan_6-exohydrolase_{ECO:0000312| EMBL:CAD48404.1};TMhmm_ ExpAA:22.73	105	731	507	1.3	0.4	0.5	3.2	2.7	–0.96
PC-3p-777_32390	TTTTTAACAATCTCTTTTCCAA	EL10Ac9g21076	Putative_disease_resistance_protein_RGA4	105	731	507	4.4	0.0	0.0	14244	360.7	–0.94
PC-3p-777_32390	TTTTTAACAATCTCTTTTCCAA	EL10Ac9g21401	Protein_FLOWERING_LOCUS_T	105	731	507	32.9	13.8	13.2	2.4	2.5	–0.93
PC-3p-777_32390	TTTTTAACAATCTCTTTTCCAA	EL10Ac9g21815	Far_upstream_element-binding_protein_3; TMhmm_ExpAA:43.56	105	731	507	2.6	0.8	0.7	3.2	3.8	–0.91
PC-5p-178120_466	AACGTCGGGCCTGGACGTTCGGGC	EL10Ac2g02731	Probable_carboxylesterase_17	0	21	10	86.9	34.3	33.3	2.5	2.6	–0.84
PC-5p-3041_10997	GGTAAGAGATTGTTAAAAATT	EL10Ac2g03607	ABC_transporter_D_family_member_2,_chloroplastic; TMhmm_ExpAA:94.97	44	217	273	8.1	3.7	2.6	2.2	3.2	–1.00
PC-5p-3041_10997	GGTAAGAGATTGTTAAAAATT	EL10Ac4g08705	Chaperone_protein_ClpB1	44	217	273	11.0	4.9	4.7	2.2	2.3	–0.98
PC-5p-3041_10997	GGTAAGAGATTGTTAAAAATT	EL10Ac8g18356	Acid_phosphatase_1;SignalP:0.911	44	217	273	13.7	6.0	6.6	2.3	2.1	–0.95
PC-5p-3041_10997	GGTAAGAGATTGTTAAAAATT	EL10Ac9g21893	LRR_receptor-like_serine/threonine-protein_kinase_FLS2;SignalP:0.902; TMhmm_ExpAA:23.41	44	217	273	2.1	0.7	1.0	3.1	2.1	–0.90
PC-5p-73236_1013	AATAGGGCTAAAAGTCGTTGC	EL10Ac3g05165	Caffeic_acid_3-O-methyltransferase	0	10	11	16.7	8.1	10.3	2.1	1.6	–0.94
PC-5p-9987_4515	TCCGGCCAGGACGTTCTGCCC	EL10Ac3g06964	Leucine-rich_repeat_receptor-like_kinase_protein_FLORAL_ORGAN_NUMBER1	3	48	66	22.8	9.9	6.8	2.3	3.4	–1.00

*A strong negative correlation (<–0.80 to –1.00) indicates higher abundance of a specific sncRNA and lower expression of corresponding sncRNA target sugar beet gene/s. Data are Mean of 3–5 biological replicates.*

**TABLE 4:** Sugar beet miRNAs that showed < 2-fold expression in the apical leaves of BCTV resistant (13, 4) vs. susceptible lines (19) at 6 dpi and effect on corresponding sugar beet target gene expression.

miRNA	Sequence	Gene ID	Gene annotation	miRNA abundance (FPKM)			Target gene expression (FPKM)			Fold change (FPKM)		Correlation coefficient
							
				Line 19	Line 13	Line 4	Line 19	Line 13	Line 4	Line 13 vs Line 19	Line 4 vs Line 19	
ath-miR160a-5p_R-1	TGCCTGGCTCCCTGTATGCC	EL10Ac1g01085	15-cis-phytoene_desaturase,_chloroplastic/chromoplastic	399	185	208	0.0	7.3	6.0	462.7	376.2	–1.00
ath-miR160a-5p_R-1	TGCCTGGCTCCCTGTATGCC	EL10Ac2g02632	Putative_disease_resistance_RPP13-like_protein_1	399	185	208	0.1	1.5	1.0	24.8	15.4	–0.95
ath-miR160a-5p_R-1	TGCCTGGCTCCCTGTATGCC	EL10Ac7g17778	Probable_LRR_receptor-like_serine/threonine-protein_kinase_At1g56140;SignalP:0.916;TMhmm_ExpAA:25.02	399	185	208	2.6	45.1	39.4	17.2	15.0	–1.00
ath-miR160a-5p_R-1	TGCCTGGCTCCCTGTATGCC	EL10Ac8g19248	MLP-like_protein_31	399	185	208	37.5	265.0	176.3	7.1	4.7	–0.96
bra-miR164e-3p_1ss14CT	CACGTGCTCCCCTTCTCCAAC	EL10Ac2g02793	Putative_disease_resistance_RPP13-like_protein_1	16	2	3	0.1	2.1	2.1	30.1	31.4	–0.99
bra-miR164e-3p_1ss14CT	CACGTGCTCCCCTTCTCCAAC	EL10Ac3g06581	Protein_FAR1-RELATED_SEQUENCE_5	16	2	3	0.0	1.6	0.9	131.5	78.5	–0.94
csi-miR164a-3p_2ss9CT10TG	CATGTGCCTGTCTTCCCCATC	EL10Ac2g04621	Protein_GAMETE_EXPRESSED_3; SignalP:0.516;TMhmm_ExpAA:45.83	6841	2470	3094	0.3	3.9	4.2	12.8	13.7	–0.98
csi-miR164a-3p_2ss9CT10TG	CATGTGCCTGTCTTCCCCATC	EL10Ac9g21321	Heterodimeric_geranylgeranyl_pyrophosphate_synthase_small_subunit,_chloroplastic	6841	2470	3094	29.9	63.7	65.4	2.1	2.2	–0.98
PC-3p-431909_166	AGTAGGCTAAACTCTCCTCCTCCC	EL10Ac3g05903	hypothetical_protein; TMhmm_ExpAA:47.05	14	2	1	24.1	56.9	66.0	2.4	2.7	–0.99
PC-3p-452967_156	TCCAGCCCTCCCCCCTACAAC	EL10Ac5g12946	Pentatricopeptide_repeat-containing_protein_PNM1,_mitochondrial	10	0	3	19.2	54.8	44.6	2.9	2.3	–1.00
PC-3p-50231_1360	TCCTGGCCGAACGATCGCCCC	EL10Ac1g01374	Serine/threonine-protein_phosphatase_7_long_form_homolog	71	31	28	0.0	2.1	2.6	20983.2	25616.2	–0.99
PC-3p-539842_121	CTACCCCTACCCCCGCCCTCTATC	EL10Ac6g14637	Heat_shock_70_kDa_protein_6,_chloroplastic	20	2	3	0.0	3.5	5.6	35208.2	56428.3	–0.91
PC-3p-909715_50	AATCTACTGTTTGGATGGCATGGA	EL10Ac4g09965	Probable_WRKY_transcription_factor_34	7	2	0	1.6	4.7	4.4	2.9	2.7	–0.93
PC-5p-205203_404	ATCCACCATGGACGCCCCACTACC	EL10Ac1g01085	15-cis-phytoene_desaturase,_chloroplastic/chromoplastic	27	1	1	0.0	7.3	6.0	462.7	376.2	–0.98
PC-5p-725678_75	AACCCGATCCATCTGTTCACCCGG	EL10Ac8g20096	Leucine-rich_repeat_receptor-like_serine/threonine-protein_kinase_BAM1	12	1	0	0.2	1.8	1.2	9.3	6.1	–0.89
PC-5p-98300_796	AGTAGGGCTGCGCTTGTAGAAAAG	EL10Ac5g12755	Thaumatin-like_protein;SignalP:0.892;TMhmm_ExpAA:29.17	28	15	6	4.7	12.3	12.8	2.6	2.7	–0.94
ptc-MIR7836-p3_2ss9CA20AC	CCACACCTACCACCCAAACCCC	EL10Ac7g16970	Pumilio_homolog_6,_chloroplastic	24	7	5	0.0	0.0	0.0	52.8	162.3	–0.81
ptc-MIR7836-p3_2ss9CA20AC	CCACACCTACCACCCAAACCCC	EL10Ac8g20096	Leucine-rich_repeat_receptor-like_serine/threonine-protein_kinase_BAM1	24	7	5	0.2	1.8	1.2	9.3	6.1	–0.88

*A strong negative correlation (<–0.80 to –1.00) indicates higher abundance of a specific sncRNA and lower expression of corresponding sncRNA target sugar beet gene/s. Data are Mean of 3–5 biological replicates.*

### Leaf Carbohydrate Contents Are Altered in the S vs. R Lines

**FIGURE 5:**
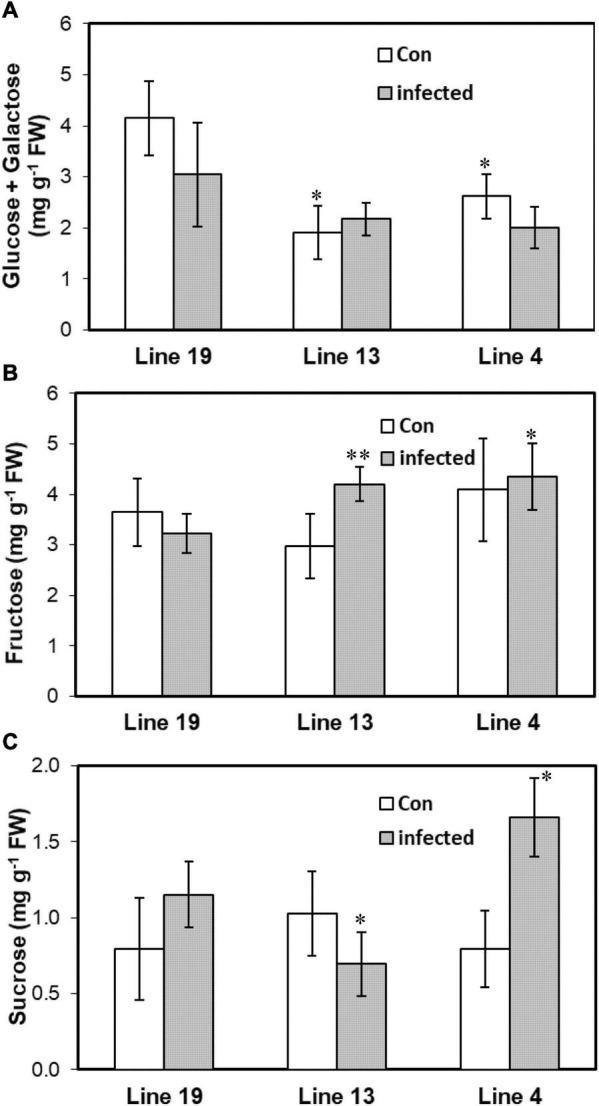
Leaf carbohydrate contents vary in the BCTV susceptible (Line 19; S) vs. resistant (Line 13 and Line 4; R) sugar beet genotypes. Cellular content of **(A)** glucose + galactose, **(B)** fructose, and **(C)** sucrose at 6 dpi in the uninfected control (Con) and inoculated plants. Data are Mean ± SE of 4–6 biological replicates (**P ≤ 0.05 and *P ≤ 0.1 between susceptible vs. resistant genotypes, Student’s *t*-test).

As miRNAs targeted genes that were highly associated with carbohydrate metabolism, we therefore investigated leaf carbohydrate contents in control and BCTV infected plants of the S and R lines. Cellular content of glucose + galactose was lower (37–54%) in the control plants of the R lines (vs. S) without any change among the lines following infection (Figure 5). Fructose on the other hand increased by 30–35% only in the infected plants of the R lines. No specific trend in leaf sucrose content was observed within R lines post BCTV infection. Sucrose content decreased by 40% in the line 13 (R) whereas, line 4 (R) showed a 44% increase in sucrose content following infection.

### Sugar Beet miRNAs Target BCTV Genes

We also investigated if any sugar beet miRNAs targeted BCTV genes and if higher expression of specific miRNAs have any effect on target viral gene expression. A complete list of miRNAs and their potential BCTV target genes are listed in Table 5. At 2 dpi miRNAs which were moderately expressed, detected only in the R lines and reduced BCTV target gene expression by >2 to 4-fold include PC-3p-28452_2099 and PC-3p-63465_1134. The miRNAs that showed high expression (FPKM 5,000–14,000) at 2 dpi and reduced target gene expression by 3 to 4-fold were ath-miR172a_1ss1AC and ath-miR162a-3p. At 6 dpi only two miRNAs were detected in sugar beet lines that showed moderate/low expression and potentially targeted BCTV genes. We found another miRNA, PC-3p-24046_2377 that showed 10 bp matches with the BCTV origin of replication and were present at both 3 dpi and 6 dpi only in the R lines. In general, we found a 10–12 bp matches between sugar beet miRNAs and their corresponding BCTV target genes and most of these sequence identities were at the seed region of the miRNAs. A majority of the sugar beet miRNAs targeted BCTV genes were associated with capsid protein (V1) and rolling circle replication initiator protein (C1).

**TABLE 5:** Relative abundance of sugar beet miRNAs and their potential targets in the BCTV genome (KX867019.1; BCTV-Svr strain).

Days post infection (dpi)	Sugar beet miRNA name	miRNA abundance (FPKM)			Percent identity	Matches	Start	Stop	BCTV target gene	e-value	Average BCTV mRNA reads/sample		
		Line 19	Line 13	Line 4							Line 19	Line 13	Line 4
2	PC-3p-28452_2099	0	14	51	100	10	1,039	1,030	V1	0.044	78	18	26.6
	ath-miR172c_R + 1	255	90	226	100	12	2,459	2,448	C1	0.004	11.25	3.6	2.6
	ath-miR172a_1ss1AC	11,258	5,221	9,922	100	11	2,459	2,449	C1	0.012	11.25	3.6	2.6
	PC-3p-24046_2377	0	30	24	100	10	9	18	Ori	0.048	na	na	na
	ath-miR162a-3p	7,629	9,976	14,859	100	10	1,052	1,043	V1	0.044	78	18	26.6
	bna-miR172a_R + 3_1ss1AC	90	22	42	100	11	2,459	2,449	C1	0.015	11.25	3.6	2.6
	PC-3p-63465_1134	0	39	23	100	10	1,212	1,203	V1	0.056	78	18	26.6
	PC-3p-247893_330	0	8	2	100	10	1,760	1,769	C1 UTR	0.052	na	na	na
6	PC-3p-24046_2377	0	30	24	100	10	18	9	Ori	0.048	na	na	na
	PC-5p-139992_586	0	8	6	100	10	1,203	1,212	V1	0.056	135,007	146.2	126.6

*Data are Mean of 3–5 biological replicates. V1, capsid protein; C1, rolling circle replication initiator protein; Ori, origin of replication; UTR, untranslated region.*

### BCTV Derived sncRNAs Modulate Sugar Beet Gene Expression

Virus derived sncRNAs were investigated by aligning sequencing reads generated from this experiment to the BCTV genome sequences. The mock, or non-infected samples did not contain reads which aligned to the virus genome, indicating no presence of the virus. The infected samples were variable in regard to the abundance of reads and representation of putative virus sncRNA species. RNA was recovered for 1, 2, and 6 dpi with BCTV. Time and inoculation were large factors in the abundance of virus transcripts. No reads mapped to the virus genome from the non-inoculated samples (data not presented) were detected. Due to low viral RNA reads at 1 and 2 dpi, only data from 6 dpi are presented here.

**FIGURE 6:**
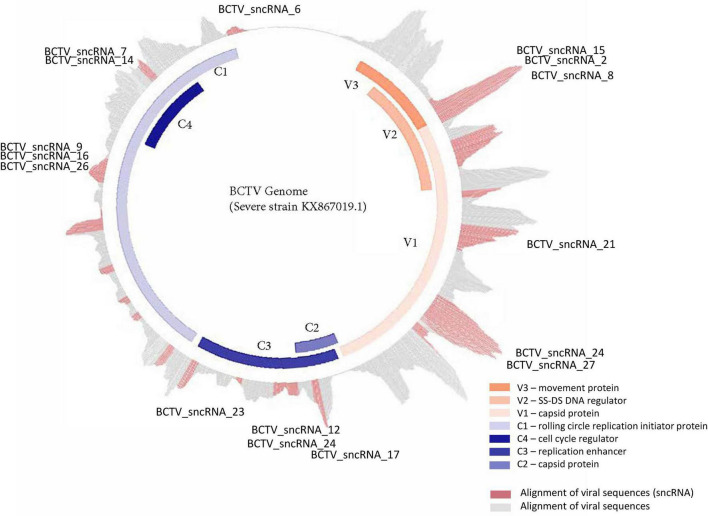
Distribution and relative abundance of small non-coding RNAs originating from BCTV during its interaction with sugar beets.

Reads that mapped to any of the four virus strain genomes produced a total of 30 putative sncRNA sequences. These unique sncRNA sequences contained a core motif which showed significant homology to the sugar beet cDNA sequences. The core sequences were 18–22 nt long apart from BCTV_sncRNA 4, which contained a core sequence length of 26 nt (Table 6). Reads that generated 18–22 nt core sequence were longer (31–41nt). This exceeded the size expected from a traditional siRNA or virus-derived siRNA (vsiRNA) but included a core sequence whose reverse complement showed 100% sequence identity to the host genome and defined as a putative sncRNA (Table 6). For visualization purposes, only sncRNA alignments to the BCTV Severe strain CTS06-021 genome were used (Figure 6). This reduced the complexity of the multi fasta alignments and demonstrated relative distribution of the putative sncRNAs. Correlation analysis between sncRNA abundance and corresponding sugar beet target gene expression (FPKM) helped to validate their potential role in BCTV resistance in sugar beet. The BCTV sncRNAs that showed highest to moderate abundance were BCTV_sncRNA 4, 18, and 19 and had the most down-regulation (1.5 to 2-fold) of target sugar beet gene expression EL10Ac1g01206 (LRR), EL10Ac5g12605 (7-deoxyloganetic acid glucosyltransferase), and EL10Ac6g14074 (transmembrane emp24 domain containing) respectively in the S line (Table 7).

**TABLE 6:** *Beet curly top virus* (BCTV) derived sncRNAs detected in the apical leaves of infected sugar beet plants at 6 dpi and their corresponding sugar beet target genes.

BCTV sncRNA species	sncRNA sequence	Length	Percent identity to sugar beet gene targets	Sugar beet gene targets	Sugar beet gene annotation
sncRNA 1	CTGGAGGAGGAAGAAAA	18	100	EL10Ac1g00033	Nitrate_reductase_[NADH]
sncRNA 2	GTGGCCGAAGAAGAGGAG	19	100	EL10Ac1g00783	Homeobox-leucine_zipper_protein_HAT3
sncRNA 3	GCTTCATTTTCTGAGTTA	19	100	EL10Ac1g01113	Protein_TRANSPARENT_TESTA_12;TMhmm_ExpAA:220.52
**sncRNA 4**	GTTCAAAAGATTGTGATGTTGAAGG	26	92.31	EL10Ac1g01206	Leucine-rich_repeat-containing_protein_46
sncRNA 5	TATCAACCCCAAAATAT	18	100	EL10Ac1g02347	AP2-like_ethylene-responsive_transcription_factor_ANT
sncRNA 6	GGGCTCTCTTCAAATCCCC	20	100	EL10Ac2g02425	Pentatricopeptide_repeat-containing_protein_At5g08305
sncRNA 7	TTTCGGAGGAGGAAGAAAAA	21	100	EL10Ac2g02734	Cytosolic_sulfotransferase_15
sncRNA 8	CTTCAATATTTGAAGTA	18	100	EL10Ac2g04434	Auxin_transport_protein_BIG
sncRNA 9	AAAGAAGAAAGAGGAAA	18	100	EL10Ac3g06583	Zinc_finger_CCCH_domain-containing_protein_32
sncRNA 10	TTTTTCAAGAAATTGTT	18	100	EL10Ac3g06769	Pentatricopeptide_repeat-containing_protein_At4g21300
sncRNA 11	CCCAAAATATGCATCAT	18	100	EL10Ac3g07263	Putative_SWI/SNF-related_matrix-associated_actin-dependent_regulator_of_chromatin_subfamily_A_member_3-like_1
sncRNA 12	GTTGTGGTTGAATCTTT	18	100	EL10Ac3g07325	Putative_disease_resistance_protein_RGA3
sncRNA 13	TGTAGCTCTCTGGCATT	18	100	EL10Ac4g08785	Heat_shock_70_kDa_protein_16
sncRNA 14	TGCAGTGGAATTGTTTG	18	100	EL10Ac4g08848	Pentatricopeptide_repeat-containing_protein_At1g11290
sncRNA 15	AAGGAAGTGAAGAAGCT	18	100	EL10Ac4g10022	Domain_of_unknown_function_(DUF3411);TMhmm_ExpAA:54.71
sncRNA 16	ATTACATTATTAATTTT	18	100	EL10Ac4g10257	RNA_recognition_motif._(a.k.a._RRM,_RBD,_or_RNP_domain)
sncRNA 17	AAGTGGGCCCCACAGGAA	19	100	EL10Ac5g10458	Hexose_carrier_protein_HEX6;TMhmm_ExpAA:44.69
**sncRNA 18**	GCTTCTTCTTTTGAAAG	18	100	EL10Ac5g12605	7-deoxyloganetic_acid_glucosyltransferase
**sncRNA 19**	GAGATATGAACAAGAGG	18	100	EL10Ac6g14074	Transmembrane_emp24_domain-containing_protein_p24delta3;TMhmm_ExpAA:37.51
sncRNA 20	CATTTGAAGTTTGATAT	18	100	EL10Ac6g14625	DNA-directed_RNA_polymerase_subunit_beta_{ECO:0000255| HAMAP-Rule:MF_01321}
sncRNA 21	CATTTGAAGTTTGATATA	19	100	EL10Ac6g14832	Myosin_heavy_chain_kinase_B
sncRNA 22	GATGTTGAAGGAAGTAA	18	100	EL10Ac6g15173	(R,S)-reticuline_7-O-methyltransferase;TMhmm_ExpAA:20.59
sncRNA 23	AATATTGAGGAAGTCTT	18	100	EL10Ac6g15406	Putative_pentatricopeptide_repeat-containing_protein_At2g02150
sncRNA 24	AGGTTTATTGTGAAGAA	18	100	EL10Ac7g16816	UPF0554_protein_C2orf43_homolog;TMhmm_ExpAA:43.89
sncRNA 25	TGTCTGTTTACCTCCTC	18	100	EL10Ac7g16868	Casparian_strip_membrane_protein_2;TMhmm_ExpAA:113.18
sncRNA 26	ATTATACTATTATATCT	18	100	EL10Ac7g17297	hypothetical_protein
sncRNA 27	ATATTAACATATCTATT	18	100	EL10Ac8g18763	Heparanase-like_protein_3;SignalP:0.717;TMhmm_ExpAA:27.47
sncRNA 28	TTTTTCAAGACTTTCAAAAA	21	100	EL10Ac8g19534	Domain_of_unknown_function_(DUF4216)
sncRNA 29	TTGAGGAAATACCAATT	18	100	EL10Ac9g21413	MADS-box_protein_AGL24
sncRNA 30	AACTTTACTTTATTTAA	18	100	EL10Ac9g21740	Protein_RAFTIN_1A;SignalP:0.872

**TABLE 7:** Relative abundance of BCTV sncRNAs detected in the apical leaves of infected sugar beet plants at 6 dpi and effect on corresponding sugar beet target gene expression.

sncRNA Species	Gene Name	sncRNA Abundance			KDH19-17 FPKM		KDH13 FPKM		KDH14-9 FPKM		Correlation coefficient
		KDH19-17	KDH13	KDH4-9	Infected	sd	Infected	sd	Infected	sd	
BCTV_sncRNA 1	EL10Ac1g00033	87	0	0	124.29	18.94	92.85	23.87	72.41	38.50	0.92
BCTV_sncRNA 2	EL10Ac1g00783	251	0	0	0.00	0.00	0.02	0.04	0.00	0.00	–0.50
BCTV_sncRNA 3	EL10Ac1g01113	**1**	0	0	3.72	0.68	5.98	0.68	6.58	1.62	–0.98
**BCTV_sncRNA 4**	EL10Ac1g01206	**162**	5	0	24.22	1.56	30.37	3.45	34.26	15.02	–0.93
BCTV_sncRNA 5	EL10Ac1g02347	1	0	0	4.26	1.02	4.01	1.07	3.97	2.89	0.99
BCTV_sncRNA 6	EL10Ac2g02425	22	0	0	2.11	0.13	1.17	0.36	1.00	0.60	0.99
BCTV_sncRNA 7	EL10Ac2g02734	2	0	0	1.40	0.17	3.86	1.35	1.83	0.48	–0.64
BCTV_sncRNA 8	EL10Ac2g04434	1	0	0	13.02	1.01	9.97	1.85	8.85	4.23	0.97
BCTV_sncRNA 9	EL10Ac3g06583	96	0	0	22.98	2.66	21.13	3.61	23.00	1.42	0.49
BCTV_sncRNA 10	EL10Ac3g06769	26	0	0	0.62	0.32	0.49	0.13	0.40	0.16	0.92
BCTV_sncRNA 11	EL10Ac3g07263	1	0	0	4.44	0.27	4.63	0.47	3.82	1.39	0.30
BCTV_sncRNA 12	EL10Ac3g07325	1	0	0	2.80	2.03	2.89	0.46	2.89	0.45	–1.00
BCTV_sncRNA 13	EL10Ac4g08785	3	0	0	0.00	0.00	3.29	0.38	3.68	0.80	–1.00
BCTV_sncRNA 14	EL10Ac4g08848	4	0	0	8.59	0.75	10.97	1.10	8.73	2.09	–0.54
BCTV_sncRNA 15	EL10Ac4g10022	57	1	0	48.90	6.51	46.82	6.17	43.04	8.82	0.78
BCTV_sncRNA 16	EL10Ac4g10257	22	0	0	0.00	0.00	0.00	0.00	0.00	0.00	na
BCTV_sncRNA 17	EL10Ac5g10458	1	0	0	25.29	2.85	20.45	3.34	24.20	5.72	0.67
**BCTV_sncRNA 18**	EL10Ac5g12605	**23**	0	0	1.13	0.39	2.46	0.58	3.33	1.26	–0.92
**BCTV_sncRNA 19**	EL10Ac6g14074	**35**	1	0	12.91	1.00	14.65	1.59	16.20	1.34	–0.89
BCTV_sncRNA 20	EL10Ac6g14625	1	0	0	0.00	0.00	0.00	0.00	0.00	0.00	na
BCTV_sncRNA 21	EL10Ac6g14832	29	0	0	0.00	0.00	0.00	0.00	0.00	0.00	na
BCTV_sncRNA 22	EL10Ac6g15173	45	2	0	0.17	0.27	0.24	0.24	1.15	0.76	–0.59
BCTV_sncRNA 23	EL10Ac6g15406	1	0	0	3.03	0.39	3.41	0.48	3.20	0.98	–0.84
BCTV_sncRNA 24	EL10Ac7g16816	1	0	0	4.80	1.17	7.44	0.40	4.64	1.31	–0.45
BCTV_sncRNA 25	EL10Ac7g16868	7	0	0	0.00	0.00	0.00	0.00	0.00	0.00	na
BCTV_sncRNA 26	EL10Ac7g17297	2	0	0	6.05	0.66	4.97	0.59	3.96	0.83	0.88
BCTV_sncRNA 27	EL10Ac8g18763	40	1	0	5.15	0.39	2.81	0.56	2.64	1.00	1.00
BCTV_sncRNA 28	EL10Ac8g19534	22	0	0	0.00	0.00	0.00	0.00	0.00	0.00	na
BCTV_sncRNA 29	EL10Ac9g21413	6	0	0	8.41	2.27	8.98	2.10	9.24	1.63	–0.95
BCTV_sncRNA 30	EL10Ac9g21740	1	0	0	1.74	0.32	2.62	0.42	2.74	0.73	–0.99

*The sncRNAs with relatively higher abundance and that showed a strong negative correlation (<-0.80 to -1.00) between sncRNA abundance and sugar beet target gene expression are in bold. Data are Mean ± SD of 3–5 biological replicates.*

### The Susceptible Line Showed Strong Disease Symptoms for All Exposure Times

**FIGURE 7:**
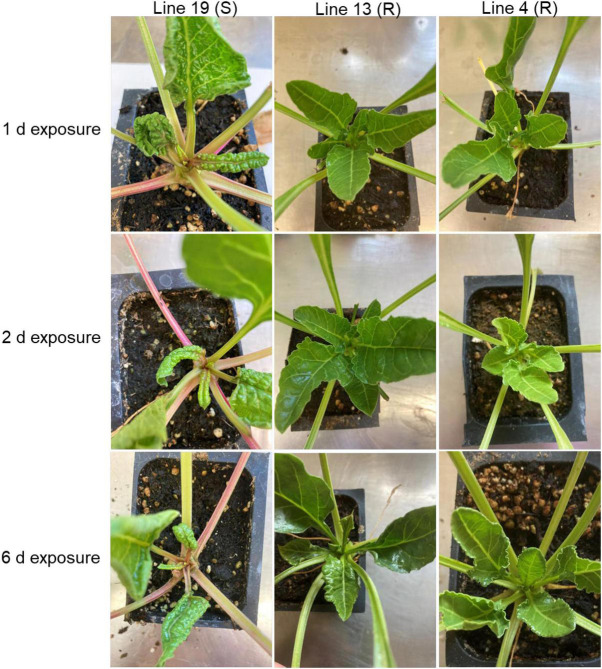
Disease symptoms in sugar beet plants that were earlier infected by BCTV. Beet curly top virus symptoms in the apical leaves at 3 weeks post BCTV inoculation in the susceptible (Line 19; S) and resistant (Line 13 and Line 4; R) sugar beet lines that were exposed to viruliferous beet leaf hoppers for 1 d, 2 d, and 6 d durations.

Both control and BCTV infected plants were evaluated for disease symptoms after three weeks post inoculation. The S line from all BCTV exposure times (1, 2, and 6 days) showed strong BCTV symptoms (3 weeks post inoculation) such as swelling of veins, leaf thickening, and leaf curling predominantly in the apical leaves whereas, the R lines had minimal to no disease symptoms at this time (Figure 7). The control plants that were kept in separate cages without exposure to any BLH did not show any disease symptoms (Supplementary Figure 4). Viral load was determined through RT-PCR estimation of the BCTV *CP* gene expression with respect to the expression of sugar beet house-keeping gene, glutamine synthetase, in BCTV inoculated plants. In general, the infected plants showed BCTV *CP* gene expression which had relatively higher signal strength in the S line (vs. R lines) at early time points and the trend was similar at 6 dpi though the overall signal strength of the *CP* gene expression were relatively higher compared to the early time points (Supplementary Figures 5–7). The sugar beet housekeeping gene showed strong expression across all samples.

## Discussion

Plant responses to biotic and abiotic stresses are mediated by diverse molecular and metabolic mechanisms. Small RNAs in this regard are known to play a key regulatory role through modulation of expression of host genes or downregulating important pathogen genes and vice versa during cross-kingdom RNAi (Huang et al., 2019). The work presented here demonstrates a global regulation of gene expression by both host and pathogen derived sncRNAs leading to sugar beet resistance or susceptibility against BCTV.

### Resistant Lines Showed Differential Expression of miRNAs vs. Susceptible Line

The DE miRNAs belonged to different categories including miRNAs that are conserved in plants and miRNAs unique to sugar beets. Upregulation of specific genes (>70 to 200-fold) such as EL10Ac2g03749 (transcription factor) and EL10Ac5g12641 (long chain acyl-coA synthetase) in the S line (vs. R) at 2 dpi/6 dpi (Tables 1, 3) due to downregulation of corresponding miRNAs raises the question if such higher expression of target genes in the S line contribute to susceptibility. On the other hand, downregulation of specific miRNAs such as ath-miR396a-5p, cas-miR172a-5p_3ss2_TC3GA9AT, and bra-miR164e-3p_1ss14CT (Tables 2, 4) in the R lines at 2 dpi/6 dpi, upregulate (∼2 to 30-fold) the expression of key target genes such as EL10Ac5g11116 (chaperone protein), EL10Ac3g05363 (ABC transporter), and EL10Ac2g02793 (putative disease resistance RPP13-like protein) respectively in the R lines (vs. S line). These types of genes are well known in plants for their contribution in disease resistance (Cheng et al., 2018; Ul Haq et al., 2019; Devanna et al., 2021). The data presented here might indicate evolutionary advantage in the R lines where genomic/allelic variations lead to the regulation of key miRNAs that contribute to host plant resistance during plant–virus interaction. Presence of specific miRNAs only in the R lines (Tables 1, 3) also suggest that these resistance traits could be purely due to the presence or absence of regulatory elements in the genome. Future comparative genome analysis of the S and R lines (Galewski and Eujayl, 2021) in context to the presence/absence of specific miRNAs will provide better insights on BCTV resistance at the genome level and could possibly contribute to marker development for resistance traits. Besides specific sugar beet miRNA targeting a specific sugar beet gene, it is also evident that in some cases a single miRNA targeting multiple genes and modulating their expression (Tables 1–4). In plants, optimization of cleavage efficiency has been prioritized over maximization of sequence complementarity between miRNAs and their targets during miRNA evolution (Voinnet, 2009; Jones-Rhoades, 2012). This has resulted in the flexibility of miRNAs to be able to target multiple genes. In our study we see both upregulation and/or downregulation of specific ‘*R*’ genes in the resistant lines following BCTV infection. In plants, LRR and nucleotide binding (NB)-LRR immune receptor proteins are encoded by *R* genes that are involved in pathogen recognition and initiating resistance responses. Earlier studies have shown that plant miRNAs negatively regulate *R* genes through the production of *trans*-acting siRNAs (tasiRNAs) and phased siRNAs (phasiRNAs) which is suppressed during viral/bacterial infection (Zhai et al., 2011; Li et al., 2012). Up-regulation of *R* genes such as EL10Ac3g05809 (RGA2), EL10Ac2g02633 (RPP13) in the resistant lines might indicate efficient regulation of miRNAs toward resistance against BCTV.

Differentially expressed miRNAs predominantly targeted genes associated mainly with transcription, protein phosphorylation, oxidation-reduction related to gene ontology and carbohydrates, amino acid (tryptophan) metabolism in relation to pathway-enrichment (Figure 4). Many of these cellular processes, pathways, and metabolites are well known in plants for their role in stress tolerance (reviewed in Rojas et al., 2014; Castro-Moretti et al., 2020). Among different metabolites, carbohydrates have been implicated in plant immune response leading to host resistance against pathogens (reviewed in Bolouri Moghaddam and Van den Ende, 2012). Carbohydrate mediated host resistance is achieved mainly through effector-triggered immunity (ETI) and/or pathogen-associated molecular patterns (PAMP)-triggered immunity against diverse type of pathogens including viruses (reviewed in Gouveia et al., 2017). As an example, cellular contents of sucrose and fructose increased in the leaves of Arabidopsis following *Tobacco rattle virus* (TRV) inoculation at early infection stages (Fernández-Calvino et al., 2014). In our study, we saw an increase in fructose content in the leaves of R lines (vs. S) at 6 dpi and when compared between Line 13 (R) infected versus control plants. Whereas leaf sucrose content increased only in one of the R lines, Line 4, at 6 dpi but decreased in the other R line, Line 13. This may indicate genotypic difference in modulation of specific carbohydrates and no specific trend could be obtained between BCTV infection and leaf sucrose content in the sugar beet R lines (vs. S). The data presented here indicate differential regulation of specific carbohydrates in sugar beets where higher abundance might contribute to resistance, whereas others such as glucose and galactose might result in viral susceptibility. The involvement of other metabolic pathways (Figure 4B) such as tryptophan and sphingolipid metabolism observed here are also in line with host plant resistance against pathogens including viruses (Castro-Moretti et al., 2020).

### Sugar Beet Counteracts BCTV by Targeting Core Pathogenicity Related Genes

The data presented here show several fold upregulations of specific miRNAs in the sugar beet R lines (vs. S) including presence of unique miRNAs such as PC-3p-28452_2099 and PC-3p-63465 observed only in the R lines following BCTV infection (Table 5). Further bioinformatics analysis to identify if any of these sugar beet miRNAs could potentially target BCTV genes revealed that a majority of them targeted genes/genome elements associated with capsid protein gene and viral replication (Table 5). In our case we found a 10–12 bp sequence complementarity between sugar beet miRNAs and BCTV target genes/other genome elements. The majority of these sequence complementarities were either at the seed region and/or 5′/3′ regions of the miRNAs. Several attributes of miRNAs that determine the stability of miRNA-mRNA complex and the efficacy of target gene silencing include complementarity at the seed region, 5′ region, specific nucleotide positions, and more recently even beyond the seed region (Chipman and Pasquinelli, 2019; Satish et al., 2019). However, seed region complementarity has been shown to not be an absolute necessity in plants for a miRNA to target a specific gene (Chipman and Pasquinelli, 2019). Critical plant miRNA target sites in viruses vary depending upon the virus species. A >3-fold reduction of the BCTV target genes by sugar beet miRNAs in some cases suggests that they possibly play an important role in down-regulating key virus genes that contribute to pathogenesis during early infection stages. Overall viral load was lower in the R lines in comparison to the susceptible line (Supplementary Figures 5–7) along with the R lines showing significant resistance against the virus (Figure 7). The duration of exposure to the virus did not show any striking difference in disease symptoms in the S line when evaluated after 3 weeks suggest that a small viral load can rapidly replicate inside a susceptible host to produce significant disease symptoms. An interesting observation from our studies show the presence of a miRNA, PC-3p-24046_2377, only in the R lines that targets the virus origin of replication. Thus, we postulate that sequence complementarity between this miRNA and the BCTV origin of replication could inhibit viral replication by spatially competing with the viral DNA polymerase and may contribute to the reduction of viral load. A similar mode of action by miRNAs has been reported in RNA viruses where binding of miRNAs to the viral genome affected viral replication and pathogenesis (reviewed in Trobaugh and Klimstra, 2017). Our observations are in line with the earlier reports where plant derived miRNAs targeted key viral genes thereby contributing to host resistance (reviewed in Liu et al., 2017). In addition, we validated miRNA target gene expression through mRNAseq (Table 5). Future functional characterization through over-expression of some of these miRNAs described above will help in delineating their precise role in BCTV resistance.

### BCTV Derived sncRNAs Target Key Sugar Beet Genes

Alignment of RNAseq reads to the virus genome showed next generation sequencing (NGS) of sRNAs is an efficient tool to detect virus derived transcripts during early stages of plant infection. Further characterization of these transcripts revealed putative virus derived sncRNAs with the potential to interact with targets in the sugar beet host genome (Tables 6, 7). The recovery of transcripts that were between 31and 41 nt in length that align to the virus genome were of interest. Other pipelines recommended size filters to identify virus-derived siRNAs (vsiRNAs; Leonetti et al., 2021) but would have recovered no vsiRNAs in our dataset. Alternative hypotheses explaining these observations may include degraded mRNA, novel mechanism of processing, less fidelity and regulation for viral transcription or size prior to processing. In any case, it is important to mention that there could be a potential interaction between sncRNAs and host genomes due to the high sequence homology between putative sncRNA core sequences and host target genes. These matches appeared to be what we expected for siRNAs regarding length. The 31-41 nt sequences generated may suggest that the production of these sequences from virus genomes is different from what has been described in other systems. Potential modifications of these transcripts could also be forthcoming. In *Entamoeba* parasites, specific oligo-adenylated modifications of sRNA populations were observed and represent novel mechanisms of sRNA regulation (Zhang et al., 2020). Reads did span the core sequence on both sides hinting at potential degradation of virus mRNA which was captured in the sRNA fractions. High potential for interaction due to sequence homology, known siRNA match core sequence length, and transcriptional spikes in the genome suggest that these sncRNAs are produced and may regulate the interaction between virus and host (Prasad et al., 2019). When sequences were aligned to a single virus genome, for example, the Severe strain CTS06-021, only 26 sncRNA sequences were found (data not presented here). This showed both the presence and absence of different putative sncRNAs as well as differences in the homology between the sncRNA core sequences and gene targets in the host genome. Further research is needed to investigate BCTV strain specificity and their interaction with the host genome resulting in distinct resistance phenotypes. This should shed more light on how variation within host genomes as well as variation in virus strain genomes modulate host virus interactions thereby contributing to host plant resistance or susceptibility.

A population genomic survey and epidemiological approaches in the field suggest potential for functional divergence among the BCTV strains (CA/Logan, Colorado, Severe and Worland) (Strausbaugh et al., 2017). Our observations highlight a potential mechanism regulating host virus interactions involving putative sncRNAs. The approach allows for complex variables such as virus strain specificity and host genome variability to be investigated. Relative abundance of sncRNAs at different time points and differential regulation of target gene expression in the S vs. R lines suggest importance of such interactions resulting in sugar beet host susceptibility and/or resistance to the virus (Table 7). The R lines were able to maintain higher expression of key genes such as EL10Ac1g01206 (LRR), EL10Ac5g12605 (7-deoxyloganetic acid glucosyltransferase), and EL10Ac6g14074 (transmembrane emp24 domain containing) against the predominant and most effective BCTV sncRNAs. The overall lower relative abundance of virus sncRNAs especially BCTV_sncRNA 4, 18, and 19 in the R lines suggest possible resistance mechanism/s by which these sncRNAs are pacified in the resistant plants (Table 7).

## Conclusion

**FIGURE 8:**
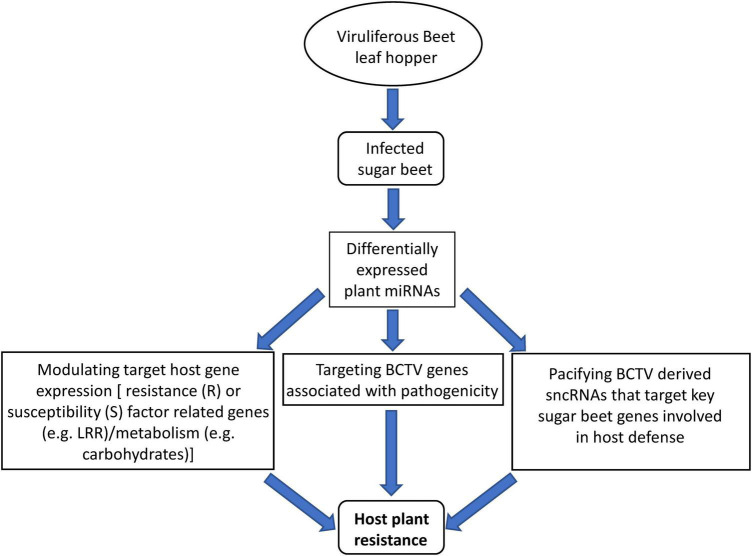
Proposed model of miRNA mediated sugar beet resistance against BCTV in the resistant genotypes.

We took a comprehensive approach by using sRNAseq, mRNAseq, analysis of target metabolites, and evaluation of symptoms to demonstrate the regulatory roles of sncRNAs and their potential contribution toward host resistance and/or susceptibility in the R and S lines respectively (Figure 8). This is the first report on the contribution of sncRNAs toward BCTV resistance in sugar beets. We have identified specific miRNAs that were DE or in some cases present only in the R lines. In the future, functional characterization of these candidate miRNAs either through overexpression in a BCTV susceptible germplasm and/or using them as molecular markers to scan diverse sugar beet genomes will help in defining their precise contribution toward BCTV resistance. Any fundamental resistance mechanism offered by these miRNAs especially their role in controlling viral replication will be invaluable for future BCTV resistance in sugar beets and possibly in other susceptible crops.

## Data Availability Statement

The original contributions presented in the study are publicly available. This data can be found here: National Center for Biotechnology Information (NCBI) BioProject database under accession numbers PRJNA764690 and PRJNA764694.

## Author Contributions

RMa and IE conceived, designed, and performed the experiments. PG, RMa, and RMi analyzed the data. RMa, PG, and RMi wrote the manuscript. EV, CS, and IE edited the draft manuscript. All authors have reviewed and approved the final manuscript.

## Author Disclaimer

Mention of trade names or commercial products in this article is solely for the purpose of providing scientific information and does not imply recommendation or endorsement by the U.S. Department of Agriculture.

## Conflict of Interest

The authors declare that the research was conducted in the absence of any commercial or financial relationships that could be construed as a potential conflict of interest.

## Publisher’s Note

All claims expressed in this article are solely those of the authors and do not necessarily represent those of their affiliated organizations, or those of the publisher, the editors and the reviewers. Any product that may be evaluated in this article, or claim that may be made by its manufacturer, is not guaranteed or endorsed by the publisher.

## Footnotes

1
http://www.miRbase.org/

2
http://rna.tbi.univie.ac.at/cgi-bin/RNAWebSuite/RNAfold.cgi

3
http://www.bioinformatics.babraham.ac.uk/projects/fastqc/

4
https://phytozome-next.jgi.doe.gov/info/Bvulgaris_EL10_1_0

5
https://github.com/BetaGenomeNinja/BCTV_sncRNA

## References

[B1] Altschul S. F. Gish W. Miller W. Myers E. W. Lipman D. J. (1990). Basic local alignment search tool. *J. Mol. Biol*. 215 403–410. 10.1016/S0022-2836(05)80360-2 2231712

[B2] Anders S. Pyl P. T. Huber W. (2014). HTSeq–a python framework to work with high-throughput sequencing data. *Bioinformatics* 31 166–169. 10.1093/bioinformatics/btu638 25260700 PMC4287950

[B3] Bennett C. W. (1971). *The Curly Top Disease of Sugar Beet and Other Plants.* St. Paul, MN: American Phytopathological Society.

[B4] Bolouri Moghaddam M. R. Van den Ende W. (2012). Sugars and plant innate immunity. *J. Exp. Bot.* 63 3989–3998. 10.1093/jxb/ers129 22553288

[B5] Briddon R. W. Watts J. Markham P. G. Stanley J. (1989). The coat protein of beet curly top virus is essential for infectivity. *Virology* 2 628–633. 10.1016/0042-6822(89)90205-5 2800340

[B6] Caracuel Z. Lozano-Durán R. Huguet S. Arroyo-Mateos M. Rodríguez-Negrete E. A. Bejarano E. R. (2012). C2 from Beet curly top virus promotes a cell environment suitable for efficient replication of geminiviruses, providing a novel mechanism of viral synergism. *New Phytol.* 194 846–858. 10.1111/j.1469-8137.2012.04080.x 22404507

[B7] Castro-Moretti F. R. Gentzel I. N. Mackey D. Alonso A. P. (2020). Metabolomics as an emerging tool for the study of plant-pathogen interactions. *Metabolites* 10:52. 10.3390/metabo10020052 32013104 PMC7074241

[B8] Cheng J. Fan H. Li L. Hu B. Liu H. Liu Z. (2018). Genome-wide identification and expression analyses of *RPP13*-like genes in Barley. *BioChip. J*. 12 102–113. 10.1007/s13206-017-2203-y

[B9] Chipman L. B. Pasquinelli A. E. (2019). miRNA targeting: growing beyond the seed. *Trends Genet*. 35 215–222. 10.1016/j.tig.2018.12.005 30638669 PMC7083087

[B10] Devanna B. N. Jaswal R. Singh P. K. Kapoor R. Jain P. Kumar G. (2021). Role of transporters in plant disease resistance. *Physiol. Plant.* 171 849–867. 10.1111/ppl.13377 33639002

[B11] Eujayl I. Strausbaugh C. Lu C. (2016). Registration of sugar beet doubled haploid line KDH13 with resistance to beet curly top. *J. Plant Regist*. 10 93–96. 10.3198/jpr2015.09.0055crgs

[B12] Fernández-Calvino L. Osorio S. Hernández M. L. Hamada I. B. del Toro F. J. Donaire L. (2014). Virus-induced alterations in primary metabolism modulate susceptibility to Tobacco rattle virus in Arabidopsis. *Plant Physiol*. 6 1821–1838. 10.1104/pp.114.250340 25358898 PMC4256867

[B13] Galewski P. J. Eujayl I. (2021). A roadmap to durable BCTV resistance using long-read genome assembly of genetic stock KDH13. *Plant Mol. Biol. Rep.* 10.1007/s11105-021-01307-5

[B14] Gouveia B. C. Calil I. P. Machado J. P. B. Santos A. A. Fontes E. P. (2017). Immune receptors and co-receptors in antiviral innate immunity in plants. *Front. Microbiol*. 7:2139. 10.3389/fmicb.2016.02139 28105028 PMC5214455

[B15] Huang C. Y. Wang H. Hu P. Hamby R. Jin H. (2019). Small RNAs - big players in plant-microbe interactions. *Cell Host Microbe* 26 173–182. 10.1016/j.chom.2019.07.021 31415750

[B16] Jones-Rhoades M. W. (2012). Conservation and divergence in plant microRNAs. *Plant. Mol. Biol.* 80 3–16. 10.1007/s11103-011-9829-2 21996939

[B17] Kanehisa M. Araki M. Goto S. Hattori M. Hirakawa M. Itoh M. (2008). KEGG for linking genomes to life and the environment. *Nucleic Acids Res.* 36 D480–D484. 10.1093/nar/gkm882 18077471 PMC2238879

[B18] Kim D. Langmead B. Salzberg S. L. (2015). HISAT: a fast spliced aligner with low memory requirements. *Nat. Methods* 12 357–360. 10.1038/nmeth.3317 25751142 PMC4655817

[B19] Leonetti P. Ghasemzadeh A. Consiglio A. Gursinsky T. Behrens S. E. Pantaleo V. (2021). Endogenous activated small interfering RNAs in virus-infected Brassicaceae crops show a common host gene-silencing pattern affecting photosynthesis and stress response. *New Phytol.* 229 1650–1664. 10.1111/nph.16932 32945560 PMC7821159

[B20] Li F. Pignatta D. Bendix C. Brunkard J. O. Cohn M. M. Tung J. (2012). MicroRNA regulation of plant innate immune receptors. *Proc. Natl. Acad. Sci. U.S.A.* 109 1790–1795. 10.1073/pnas.1118282109 22307647 PMC3277104

[B21] Li H. (2013). *Aligning Sequence Reads, Clone Sequences and Assembly Contigs with BWA-MEM.* Available online at: https://arxiv.org/abs/1303.3997 (accessed May, 2021).

[B22] Li H. Handsaker B. Wysoker A. Fennell T. Ruan J. Homer N. (2009). The sequence alignment/map format and SAMtools. *Bioinformatics* 25 2078–2079. 10.1093/bioinformatics/btp352 19505943 PMC2723002

[B23] Li X. Shahid M. Q. Wu J. Wang L. Liu X. Lu Y. (2016). Comparative small RNA analysis of pollen development in autotetraploid and diploid rice. *Int. J. Mol. Sci.* 17:499. 10.3390/ijms17040499 27077850 PMC4848955

[B24] Liu S. R. Zhou J. J. Hu C. G. Wei C. L. Zhang J. Z. (2017). MicroRNA-mediated gene silencing in plant defense and viral counter-defense. *Front. Microbiol.* 8:1801. 10.3389/fmicb.2017.01801 28979248 PMC5611411

[B25] Majumdar R. Rajasekaran K. Cary J. W. (2017). RNA Interference (RNAi) as a potential tool for control of mycotoxin contamination in crop plants: concepts and considerations. *Front. Plant Sci.* 8:200. 10.3389/fpls.2017.00200 28261252 PMC5306134

[B26] Martin M. (2011). Cutadapt removes adapter sequences from high-throughput sequencing reads. *EMBnet. J*. 17 10–12. 10.14806/ej.17.1.200

[B27] Panella L. Kaffka S. K. Lewellen R. T. McGrath J. M. Metzger M. S. Strausbaugh C. A. (2014). “Sugarbeet,” in *Yield Gains in Major U.S. Field Crops*, eds Smith S. Diers B. Specht J. Carver B. (Madison, WI: Soil Science Society of America), 357–396. CSSA Special Publication 33.

[B28] Pertea M. Pertea G. M. Antonescu C. M. Chang T. C. Mendell J. T. Salzberg S. L. (2015). StringTie enables improved reconstruction of a transcriptome from RNA-seq reads. *Nat. Biotechnol.* 33 290–295. 10.1038/nbt.3122 25690850 PMC4643835

[B29] Prasad A. Sharma N. Muthamilarasan M. Rana S. Prasad M. (2019). Recent advances in small RNA mediated plant-virus interactions. *Crit. Rev. Biotechnol.* 39 587–601. 10.1080/07388551.2019.1597830 30947560

[B30] Ramesh S. V. Yogindran S. Gnanasekaran P. Chakraborty S. Winter S. Pappu H. R. (2021). Virus and viroid-derived small RNAs as modulators of host gene expression: molecular insights into pathogenesis. *Front. Microbiol.* 11:614231. 10.3389/fmicb.2020.614231 33584579 PMC7874048

[B31] Riolo G. Cantara S. Marzocchi C. Ricci C. (2020). miRNA targets: from prediction tools to experimental validation. *Methods Protoc.* 4:1. 10.3390/mps4010001 33374478 PMC7839038

[B32] Robinson M. D. McCarthy D. J. Smyth G. K. (2010). edgeR: a bioconductor package for differential expression analysis of digital gene expression data. *Bioinformatics* 26 139–140. 10.1093/bioinformatics/btp616 19910308 PMC2796818

[B33] Rojas C. M. Senthil-Kumar M. Tzin V. Mysore K. S. (2014). Regulation of primary plant metabolism during plant-pathogen interactions and its contribution to plant defense. *Front. Plant Sci.* 5:17. 10.3389/fpls.2014.00017 24575102 PMC3919437

[B34] Satish D. Mukherjee S. K. Gupta D. (2019). PAmiRDB: a web resource for plant miRNAs targeting viruses. *Sci. Rep.* 9:4627. 10.1038/s41598-019-41027-1 30874591 PMC6420685

[B35] Srivastava S. N. (2004). “Management of sugarbeet diseases,” in *Management of Fruits and Vegetable Diseases*, ed. Mukerji K. G. (Dordrecht: Springer), 1.

[B36] Strausbaugh C. A. Eujayl I. A. Foote P. (2010). Seed treatments for the control of insects and diseases in sugarbeet. *J. Sugar Beet Res.* 47 105–125. 10.5274/jsbr.47.3.105

[B37] Strausbaugh C. A. Eujayl I. A. Panella L. W. (2013). Interaction of sugar beet host resistance and Rhizoctonia solani AG-2-2 IIIB strains. *Plant Dis.* 97 1175–1180. 10.1094/PDIS-11-12-1078-RE 30722409

[B38] Strausbaugh C. A. Eujayl I. A. Wintermantel W. M. (2017). Beet curly top virus Strains associated with sugar beet in Idaho, Oregon, and a Western U.S. Collection. *Plant Dis.* 101 1373–1382. 10.1094/PDIS-03-17-0381-RE 30678603

[B39] Strausbaugh C. A. Eujayl I. A. Panella L. W. Hanson L. E. (2011). Virulence, distribution, and diversity of *Rhizoctonia solani* from sugar beet in Idaho and Oregon. *Can. J. Plant Pathol*. 33 210–226. 10.1080/07060661.2011.558523

[B40] Strausbaugh C. A. Gillen A. M. Camp S. Shock C. C. Eldredge E. P. Gallian J. J. (2007). Relationship of beet curly top foliar ratings to sugar beet yield. *Plant Dis.* 91 1459–1463. 10.1094/PDIS-91-11-1459 30780752

[B41] Strausbaugh C. A. Wenninger E. J. Eujayl I. A. (2012). Management of severe curly top in sugar beet with insecticides. *Plant Dis.* 96 1159–1164. 10.1094/PDIS-01-12-0106-RE 30727055

[B42] Strausbaugh C. A. Wenninger E. J. Eujayl I. A. (2014). Control of curly top in sugar beet with seed and foliar insecticides. *Plant Dis.* 98 1075–1080. 10.1094/PDIS-12-13-1260-RE 30708792

[B43] Teng K. Chen H. Lai J. Zhang Z. Fang Y. Xia R. (2010). Involvement of C4 protein of beet severe curly top virus (Family *Geminiviridae*) in virus movement. *PLoS One* 5:e11280. 10.1371/journal.pone.0011280 20585583 PMC2892029

[B44] Trobaugh D. W. Klimstra W. B. (2017). MicroRNA regulation of RNA virus replication and pathogenesis. *Trends Mol. Med.* 23 80–93. 10.1016/j.molmed.2016.11.003 27989642 PMC5836316

[B45] Ul Haq S. Khan A. Ali M. Khattak A. M. Gai W. X. Zhang H. X. (2019). Heat shock proteins: dynamic biomolecules to counter plant biotic and abiotic stresses. *Int. J. Mol. Sci.* 20:5321. 10.3390/ijms20215321 31731530 PMC6862505

[B46] Voinnet O. (2009). Origin, biogenesis, and activity of plant microRNAs. *Cell* 136 669–687. 10.1016/j.cell.2009.01.046 19239888

[B47] Wu H. J. Ma Y. K. Chen T. Wang M. Wang X. J. (2012). PsRobot: a web-based plant small RNA meta-analysis toolbox. *Nucleic Acids Res.* 40 W22–W28. 10.1093/nar/gks554 22693224 PMC3394341

[B48] Xu P. Wu Q. Yu J. Rao Y. Kou Z. Fang G. (2020). A systematic way to infer the regulation relations of miRNAs on target genes and critical miRNAs in cancers. *Front. Genet.* 11:278. 10.3389/fgene.2020.00278 32296462 PMC7136563

[B49] Yang X. Zhang L. Yang Y. Schmid M. Wang Y. (2021). miRNA mediated regulation and interaction between plants and pathogens. *Int. J. Mol. Sci.* 22:2913. 10.3390/ijms22062913 33805611 PMC7999934

[B50] Zhai J. Jeong D. H. De Paoli E. Park S. Rosen B. D. Li Y. (2011). MicroRNAs as master regulators of the plant NB-LRR defense gene family via the production of phased, trans-acting siRNAs. *Genes Dev.* 25 2540–2553. 10.1101/gad.177527.111 22156213 PMC3243063

[B51] Zhang H. Ehrenkaufer G. M. Hall N. Singh U. (2020). Identification of oligo-adenylated small RNAs in the parasite Entamoeba and a potential role for small RNA control. *BMC Genomics* 21:879. 10.1186/s12864-020-07275-6 33297948 PMC7724847

[B52] Zhang H. Meltzer P. Davis S. R. (2013). RCircos: an R package for Circos 2D track plots. *BMC Bioinform.* 14:244. 10.1186/1471-2105-14-244 23937229 PMC3765848

