# Supplementary material for: Regulatory Roles of Small Non-coding RNAs in Sugar Beet Resistance Against Beet curly top virus
Source: Front Plant Sci. 2022 Jan 10;12:780877. doi: 10.3389/fpls.2021.780877 (PMC8786109; doi:10.3389/fpls.2021.780877)
Supplement: Supplementary file 1 [file Data_Sheet_1.PDF]

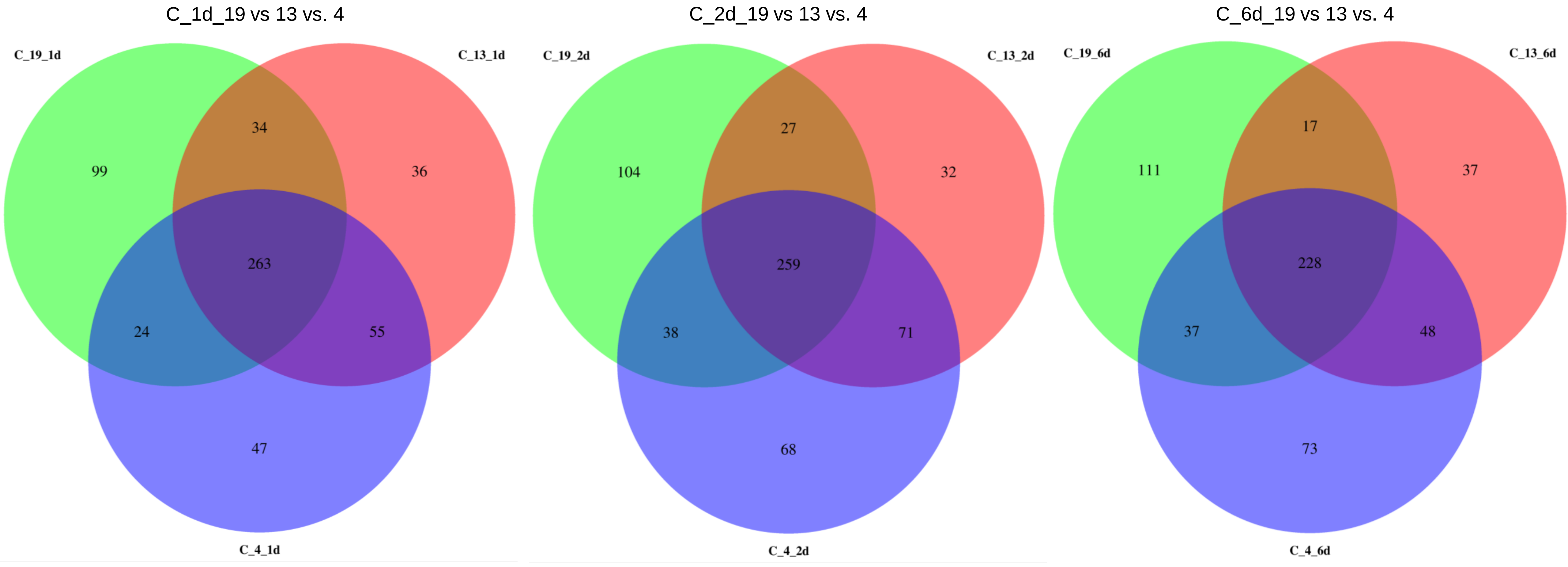

Figure S1. Venn diagrams of detected miRNAs in the apical leaves of control (uninfected; C) BCTV susceptible (Line 19; S) and resistant (Line 13 and Line 4; R) sugar beet lines at 1 d, 2 d, and 6 d.

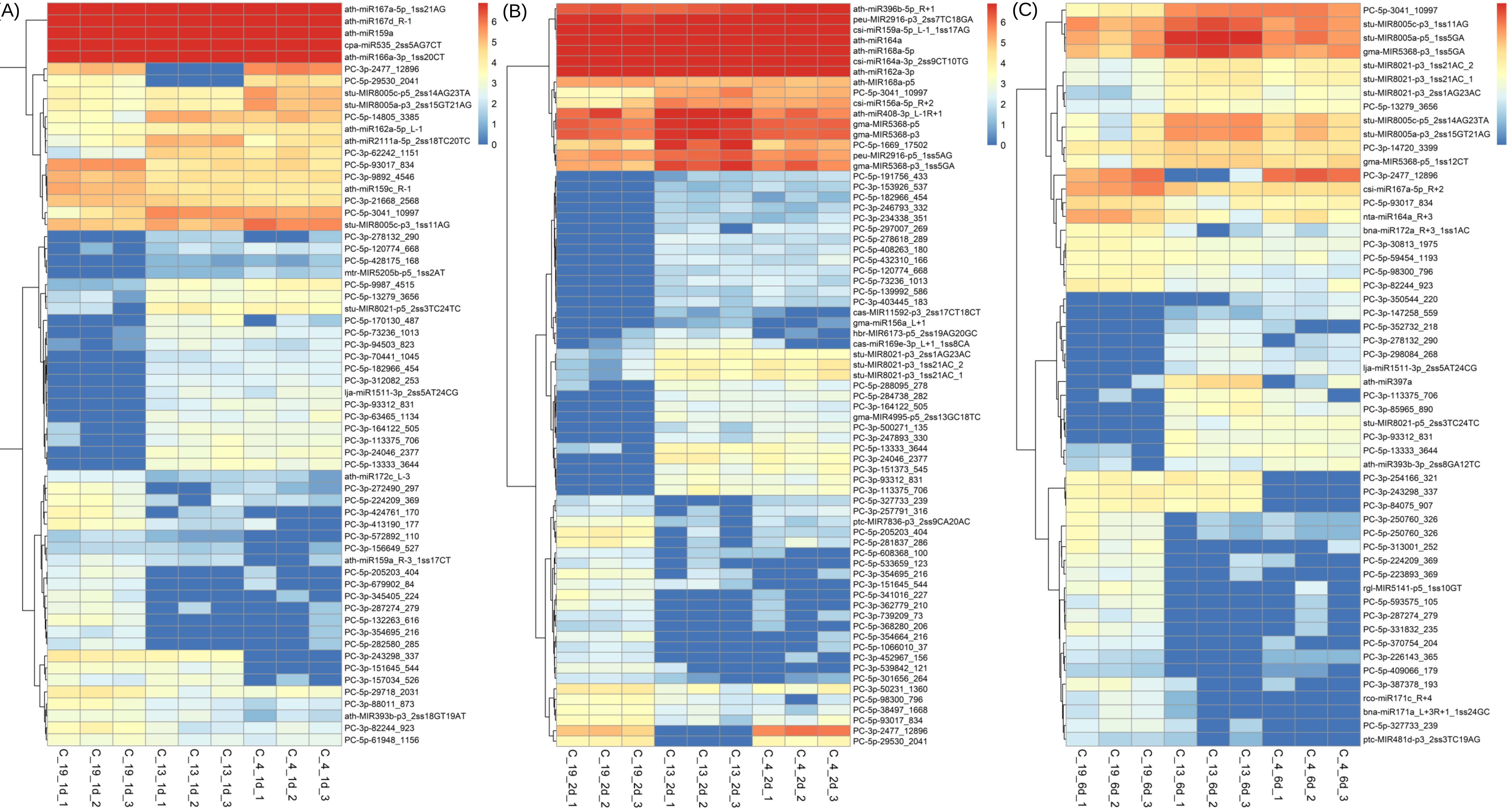

Figure S2. Heatmaps of differentially expressed (DE) miRNAs (p<0.01) in the uninfected apical leaves of BCTV susceptible (Line 19; S) and resistant (Line 13 and Line 4; R) sugar beet genotypes at (A) 1 dpi, (B) 2 dpi, and (C) 6 dpi. Data are Mean±SE of 3-5 biological replicates.

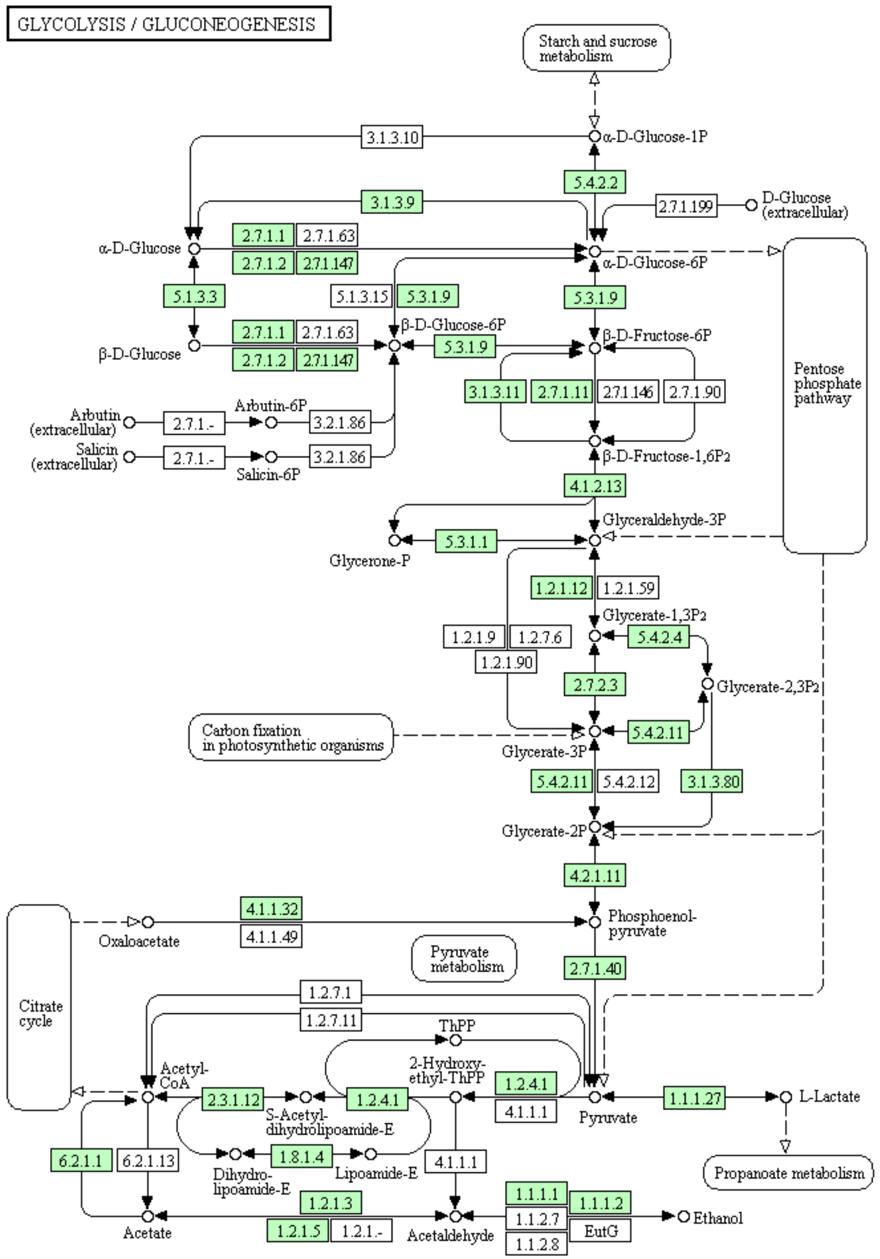

Figure S3. Differentially expressed miRNA target genes associated with carbohydrate metabolism in the leaves of sugar beet genotypes.

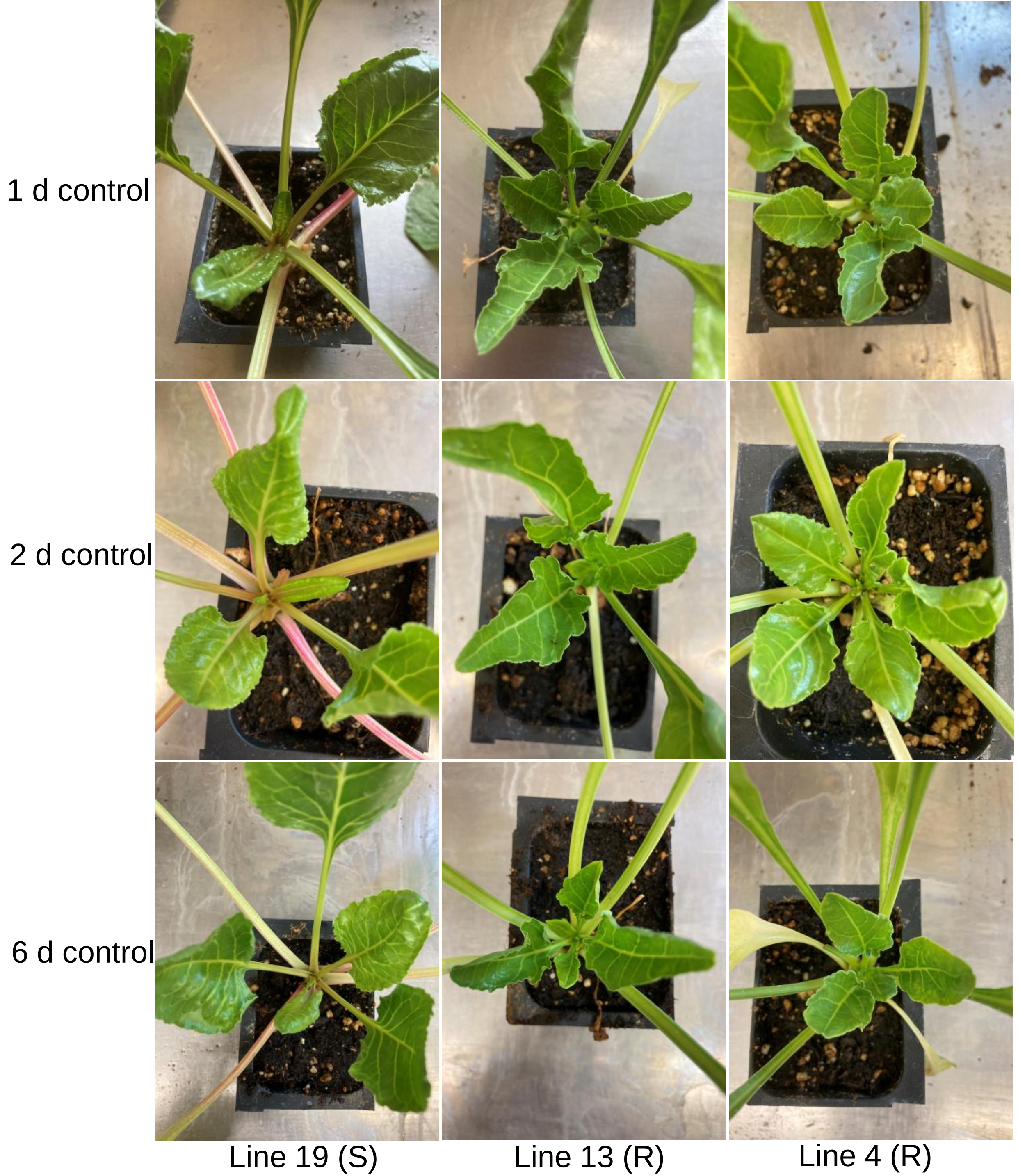

Figure S4. No disease symptoms were observed in the uninfected control sugar beet plants of the susceptible (Line 19; S) and resistant (Line 13 and Line 4; R) lines at 1 d, 2 d, and 6 d.

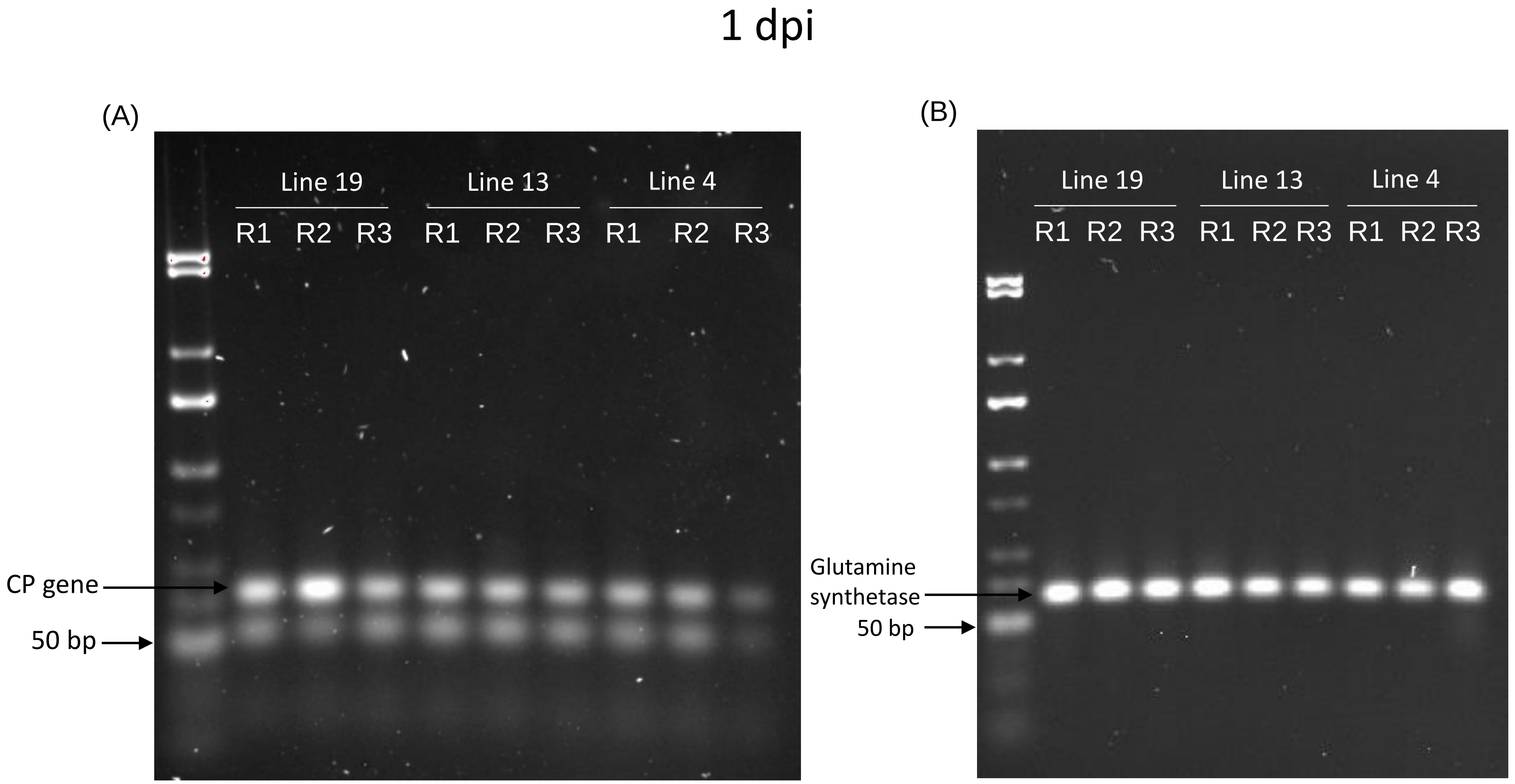

Figure S5. RT-PCR analysis of viral load in the infected samples of BCTV susceptible and resistant sugar beet genotypes at 1 dpi. (A) Expression of BCTV coat protein (CP) gene; (B) expression of sugar beet housekeeping gene, *glutamine synthetase*, in the same samples under the same thermocycling conditions. NEB TriDye™ Ultra Low Range DNA Ladder was used as a marker.

2 dpi

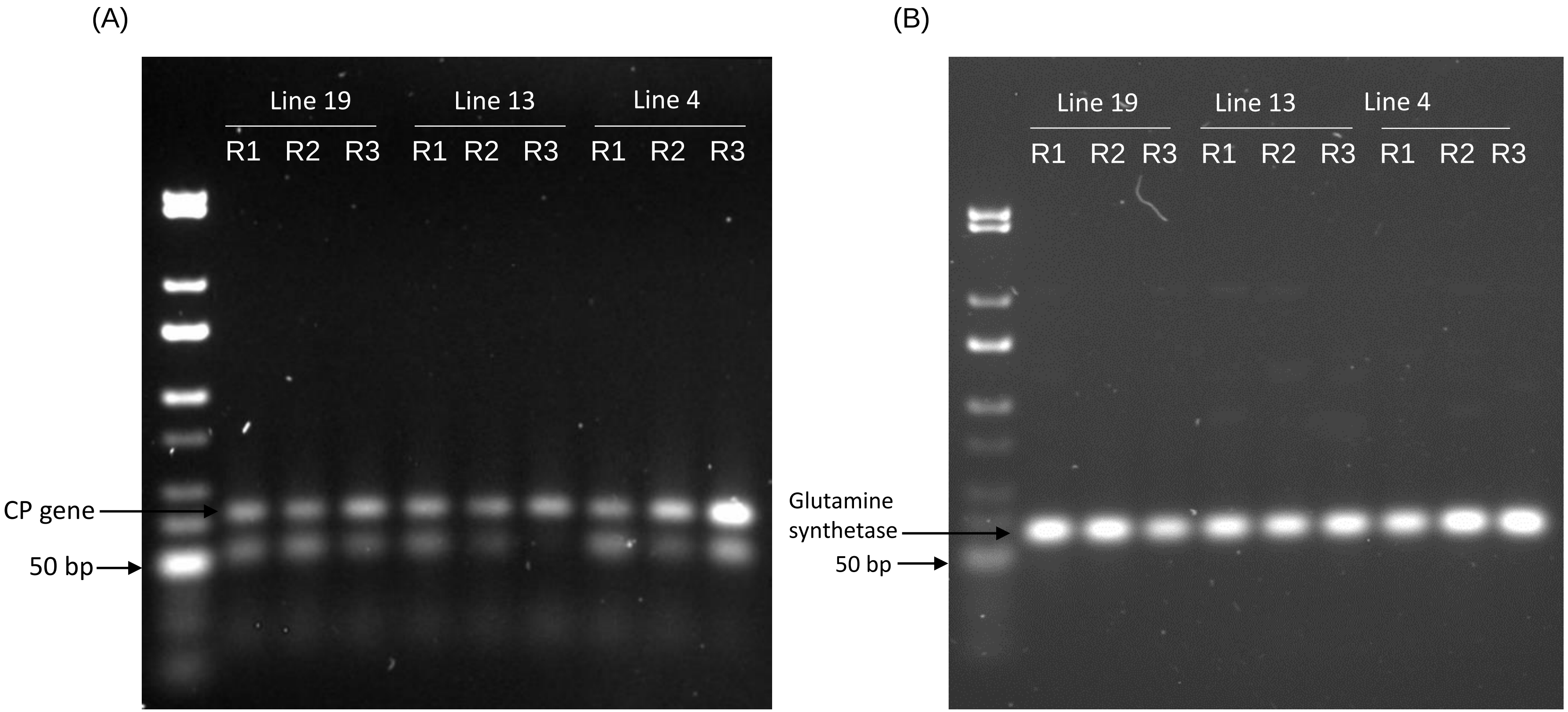

Figure S6. RT-PCR analysis of viral load in the infected samples of BCTV susceptible and resistant sugar beet genotypes at 2 dpi. (A) Expression of BCTV coat protein (CP) gene; (B) expression of sugar beet housekeeping gene, *glutamine synthetase*, in the same samples under the same thermocycling conditions. NEB TriDye™ Ultra Low Range DNA Ladder was used as a marker.

6 dpi

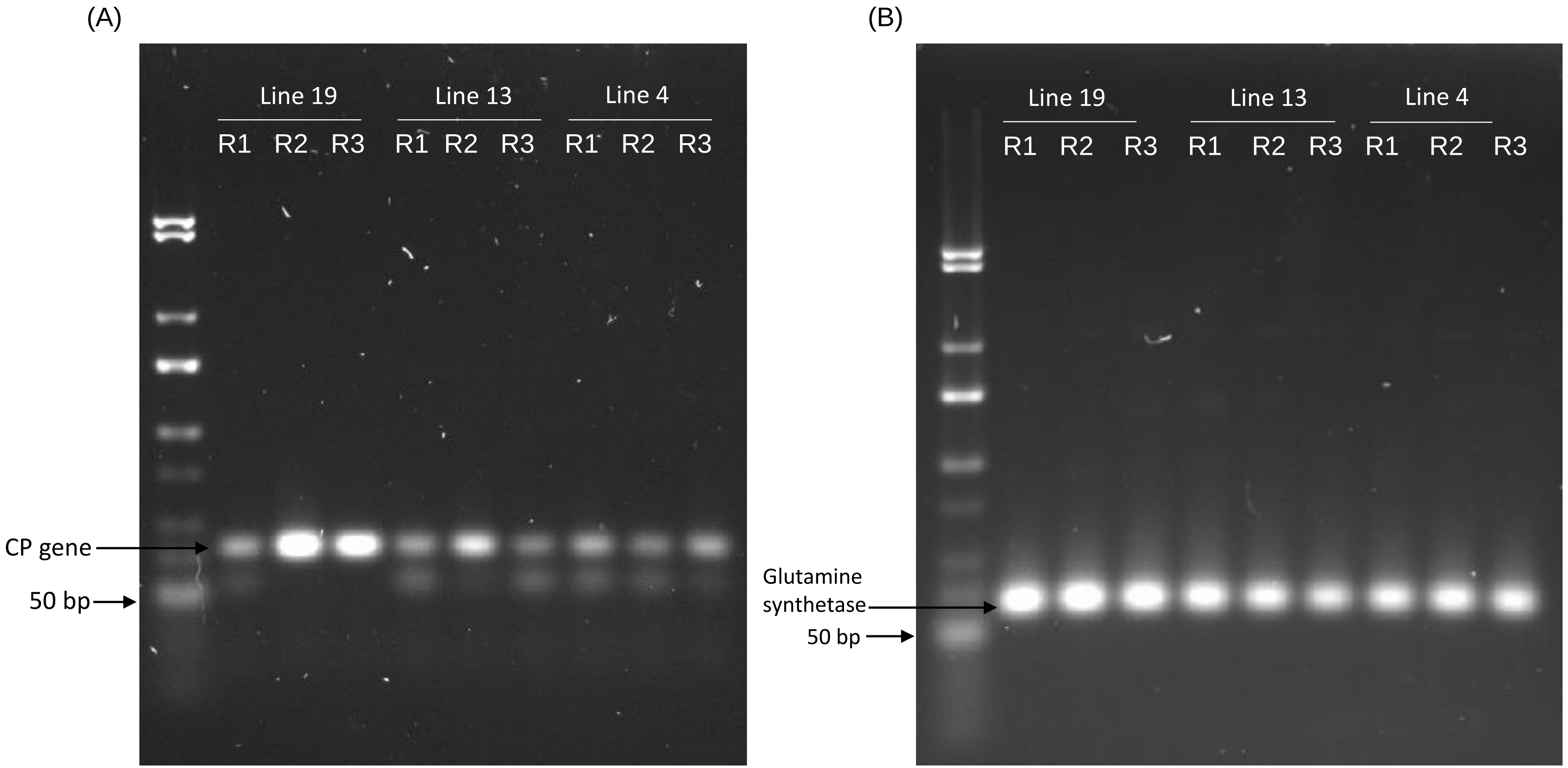

Figure S7. RT-PCR analysis of viral load in the infected samples of BCTV susceptible and resistant sugar beet genotypes at 6 dpi. (A) Expression of BCTV coat protein (CP) gene; (B) expression of sugar beet housekeeping gene, *glutamine synthetase*, in the same samples under the same thermocycling conditions. NEB TriDye™ Ultra Low Range DNA Ladder was used as a marker.
